# Ribosomal protein control of hematopoietic stem cell transformation through regulation of metabolism

**DOI:** 10.1016/j.celrep.2025.116688

**Published:** 2025-12-12

**Authors:** Bryan Harris, Dinesh K. Singh, Billy Truong, Michele Rhodes, Rachael Price, Susan Shinton, Monika Verma, Bridget Aylward, Shawn P. Fahl, Shanna R. Sprinkle, Sarah Aminov, Minshi Wang, Yong Zhang, Jaqueline Perrigoue, Rachel Kessel, Suraj Peri, Joshua West, Orsi Giricz, Jacqueline Boultwood, Andrea Pellagatti, K.H. Ramesh, Cristina Montagna, Kith Pradhan, Jeffrey W. Tyner, Brian K. Kennedy, Michael Holinstat, Ulrich Steidl, Stephen Sykes, Amit Verma, David L. Wiest

**Affiliations:** 1 Nuclear Dynamics and Cancer Program, Fox Chase Cancer Center, Philadelphia, PA 19111, USA; 2 Department of Surgery, Duke University Medical Center, Durham, NC 27710, USA; 3 Department of Biostatistics, University of North Carolina, Chapel Hill, Chapel Hill, NC 27599, USA; 4 Department of Oncology, Albert Einstein College of Medicine, Montefiore Medical Center, Bronx, NY 10461, USA; 5 Biostatistics and Bioinformatics Facility, Fox Chase Cancer Center, Philadelphia, PA 19111, USA; 6 Department of Pharmacology, University of Michigan, Ann Arbor, MI, USA; 7 Department of Internal Medicine, Division of Cardiovascular Medicine, University of Michigan, Ann Arbor, MI 48109, USA; 8 LLR Molecular Haematology Unit, NDCLS, John Radcliffe Hospital, and NIHR Biomedical Research Centre, Oxford OX3 9DU, UK; 9 Division of Hematology and Medical Oncology, Oregon Health & Science University Knight Cancer Institute, Portland, OR 97239, USA; 10 Departments of Biochemistry and Physiology, Yong Loo Lin School of Medicine, National University of Singapore, Singapore 117597, Singapore; 11 Department of Pediatrics, Washington University School of Medicine, St. Louis, MO 63110, USA; 12 These authors contributed equally; 13 Lead contact

## Abstract

We report here that expression of the ribosomal protein RPL22 is frequently reduced in human myelodysplastic syndrome (MDS) and acute myelogenous leukemia (AML), and reduced RPL22 expression is associated with worse outcomes. Mice null for Rpl22 display characteristics of an MDS-like syndrome and develop leukemia at an accelerated rate. Rpl22-deficient mice also display enhanced hematopoietic stem cell (HSC) self-renewal and obstructed differentiation potential, which arises not from reduced protein synthesis but from altered metabolism, including increased fatty acid oxidation (FAO) and a striking induction of the stemness factor Lin28b in the resulting leukemia. Lin28b promotes a substantial increase in lipid content, upon which the survival of Rpl22-deficient leukemias depends. Altogether, these findings reveal that Rpl22 insufficiency enhances the leukemia potential of HSCs through regulation of FAO and promotes leukemogenesis through Lin28b promotion of lipid synthesis.

## In brief

Harris et al. demonstrate that insufficiency of the ribosomal protein Rpl22 controls the transformation potential of hematopoietic stem cells (HSCs) not by altering protein synthesis but instead through regulating lipid metabolism, including both fatty acid oxidation and triglyceride synthesis.

## Graphical Abstract

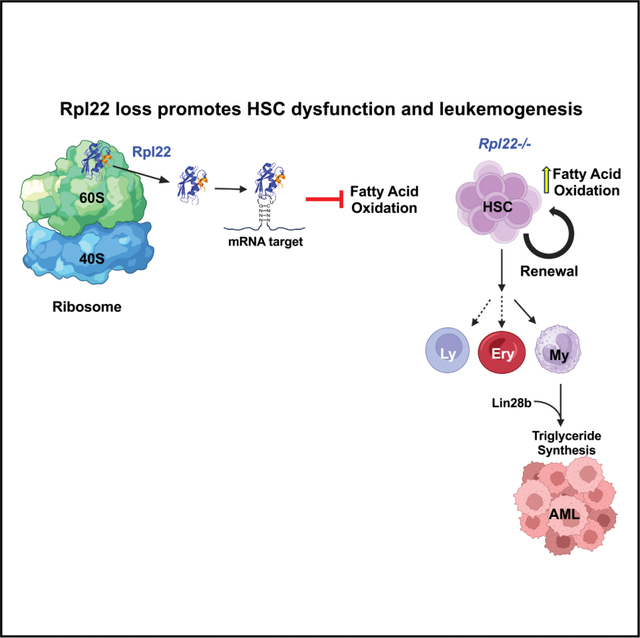

## INTRODUCTION

Ribosomopathies are a group of inherited diseases resulting from mutations or deletions of ribosomal protein (RP)-encoding genes or factors that facilitate ribosome biogenesis.^1–3^ This class of diseases is characterized by defects in hematopoiesis that often culminate in complete bone marrow failure, with many patients progressing to the development of myelodysplastic syndromes (MDSs) and acute myelogenous leukemia (AML)^4^ as a result of the aberrant function of hematopoietic stem cells (HSCs).^5^ The association between the inactivation of RP and increased risk for myeloid malignancy has been known for some time, but the mechanistic link remains unclear and controversial.^6^ Some evidence suggests that RP mutations disrupt hematopoiesis and increase cancer risk by generalized impairment of ribosome function,^7–9^ while others suggest that particular RNA-binding RPs are capable of performing specific regulatory functions.^10–12^ Indeed, *RPS14* haploinsufficiency is reported to promote the 5q− subtype of MDS through S100A8-mediated induction of p53^13^ ; however, the mechanistic link for this effect remains to be established. Several other studies have highlighted “extra-ribosomal” regulatory functions of RP involving their ability to bind to RNA targets and modulate processes, including pre-mRNA splicing and translation.^12,14,15^ These findings highlight a need to systematically investigate the contribution of RP inactivation, or reductions in their expression, to the etiology of myeloid neoplasms to gain insight into how the lost regulatory functions of RP might contribute to malignant hematopoiesis.

One RP of particular interest is Rpl22, an RNA-binding RP that binds to the 28S rRNA and is located on the exterior surface of the 60S ribosomal subunit.^16,17^ While this highly conserved RP is ubiquitously expressed in mammalian tissues, it is distinguished from many other RPs in that it is not required for ribosome biogenesis or global protein translation.^18^ Moreover, the germline ablation of the *Rpl22* gene in mice does not result in lethality or gross developmental abnormalities.^19^ Rather, Rpl22 deficiency causes selective alterations in lymphopoiesis, consistent with the loss of a regulatory function on which selected cell types are particularly dependent. Indeed, Rpl22 deficiency attenuates the development of B lymphocytes and αβ-lineage T lymphocytes in a p53-dependent manner while sparing other lineages, including γδ T cells.^19,20^ Rpl22 also regulates the emergence of embryonic HSCs by controlling the translation of *Smad1* mRNA.^21^ Finally, *RPL22* mutations and deletions have been observed in human T acute lymphoblastic leukemia (T-ALL), and Rpl22 loss promotes lymphoma development in a mouse model of T-ALL, suggesting that it functions as a tumor suppressor.^22^

The ability of Rpl22 to regulate lymphoid development and transformation, as well as fetal HSC emergence, led us to investigate whether Rpl22 also plays a role in regulating the function of adult HSCs and their potential for transformation into myeloid malignancy. Here, we show that reduced expression of *RPL22* is seen in human MDS and AML and is linked to poor outcomes. This association appears to be causal since Rpl22 loss in mice predisposes to transformation and does so not through alterations in global protein synthesis but rather through alterations in HSC metabolism and ultimately through Lin28b-mediated promotion of lipid synthesis in the resulting leukemias.

## RESULTS

### *RPL22* expression is reduced in human MDS and AML

**Figure 1.**
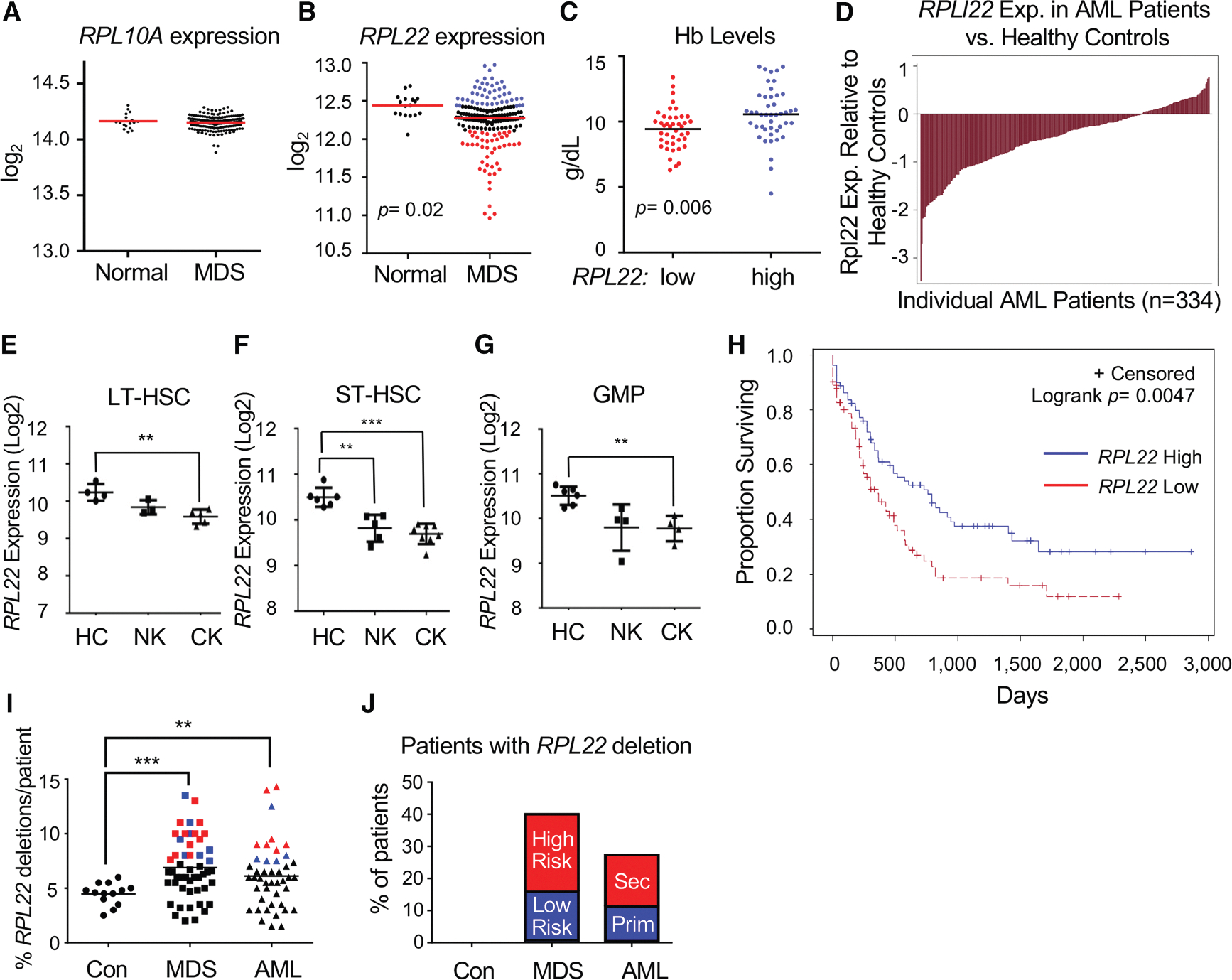
Reduced *RPL22* expression correlates with more severe MDS and AML (A and B) Expression levels of *RPL10A* and *RPL22* mRNA in CD34^+^ cells from patients with MDS (*n* = 183). (C) Hemoglobin (Hb) levels of patients with MDS with low (bottom quartile) or high (top quartile) *RPL22* expression. (D) Waterfall plot of *RPL22* expression in leukocytes from patients with AML and healthy controls enrolled in the Beat AML consortium. (E–G) *RPL22* mRNA levels in sorted (E) LT-HSCs (Lin^−^, CD34^+^, CD38^−^, CD90^+^), (F) ST-HSCs (Lin^−^, CD34^+^, CD38^−^, CD90^−^), and (G) GMP (Lin^−^, CD34^+^, CD38^+^, CD123^+^, CD45RA^+^) from bone marrow samples (*n* = 12) of patients with AML compared to age-matched healthy controls (HC; *n* = 4). Cytogenetic abnormalities are depicted as normal karyotype (NK) or complex karyotype (CK). (H) Graph of survival of patients with AML from the TCGA database, with patients with low *RPL22* expression (bottom quartile) compared to those with high expression (top quartile). The survival curve was analyzed for significance using the Mantel-Cox log rank test. (I and J) FISH analysis of deletion of the *RPL22* locus in bone marrow cells from 85 patients with MDS or AML. (I) Dot plot representing the fraction of CD34^+^ cells in which the *RPL22* locus was deleted. Patients in which the frequency of *RPL22* deletion is 3 SD greater than the mean of healthy controls are colored, with blue and red colors representing low- and high-risk MDS, respectively (middle), or primary and secondary AML, respectively (right). (J) The distribution of patients with a frequency of *RPL22* deletions greater than 3 SD of control were subdivided into low- and high-risk MDS, and primary and secondary AML and represented graphically. All graphs were analyzed for significance using the Student’s *t* test unless otherwise specified. Error bars represent SEM; **p ≤ 0.01, ***p ≤ 0.001.

To assess the changes in RP expression levels in human myeloid neoplasms, we performed gene expression analysis of CD34^+^ hematopoietic progenitor cells of 183 patients with MDS. This analysis revealed that *RPL22* is one of the most significantly reduced RPs in this disease (Figures 1A and 1B). We also observed that reduced *RPL22* expression was associated with lower hemoglobin (Hb) levels, a marker of MDS severity (Figure 1C).^23^ Since MDS often progresses to AML, we evaluated *RPL22* expression in bulk tumor from patients with AML using data obtained from the Beat AML consortium and The Cancer Genome Atlas (TCGA).^24^ Strikingly, *RPL22* expression was significantly decreased among most patients with AML compared to healthy controls (Figures 1D and S1A). We further examined *RPL22* expression levels in the immature cells from which this disease originates by stringently sorting hematopoietic stem and progenitor cell (HSPC) populations from patients diagnosed with AML (Figures 1E–1G).^25^ From these analyses, we observed a significant reduction of *RPL22* mRNA in the leukemia-initiating populations comprising long-term HSCs (LT-HSCs; Lin^−^CD34^+^CD38^−^CD90^+^), short-term HSCs (ST-HSCs; Lin^−^CD34^+^CD38^−^CD90^−^), and granulocyte-macrophage progenitors (GMPs; Lin^−^CD34^+^CD38^+^CD123^+^CD45RA^+^) of patients with higher-risk AML (associated with complex karyotype [CK]) compared to identically sorted cells from age-matched, healthy controls (Figures 1E–1G).^26,27^ Moreover, reduced expression of *RPL22* was associated with more aggressive AML, since the patients with low *RPL22* expression (bottom quartile) from the TCGA AML cohort exhibited reduced overall survival (Figure 1H). Using fluorescent *in situ* hybridization (FISH) analysis in another independent cohort of MDS/AML samples, we found that the *RPL22* locus was more frequently deleted in progenitor cells from both patients with MDS and with AML (Figure 1I). Approximately 40% of patients with MDS and 27% of patients with AML showed evidence of increased *RPL22* deletion, with more of these patients being represented in the high-risk MDS and secondary AML groups (Figure 1J).^28^ Collectively, these data indicate that *RPL22* expression is frequently reduced in patients with MDS/AML, including in their HSPCs, and that low *RPL22* expression is associated with reduced survival and thus more aggressive myeloid disease.

### Rpl22-deficient mice display an MDS-like phenotype

**Figure 2.**
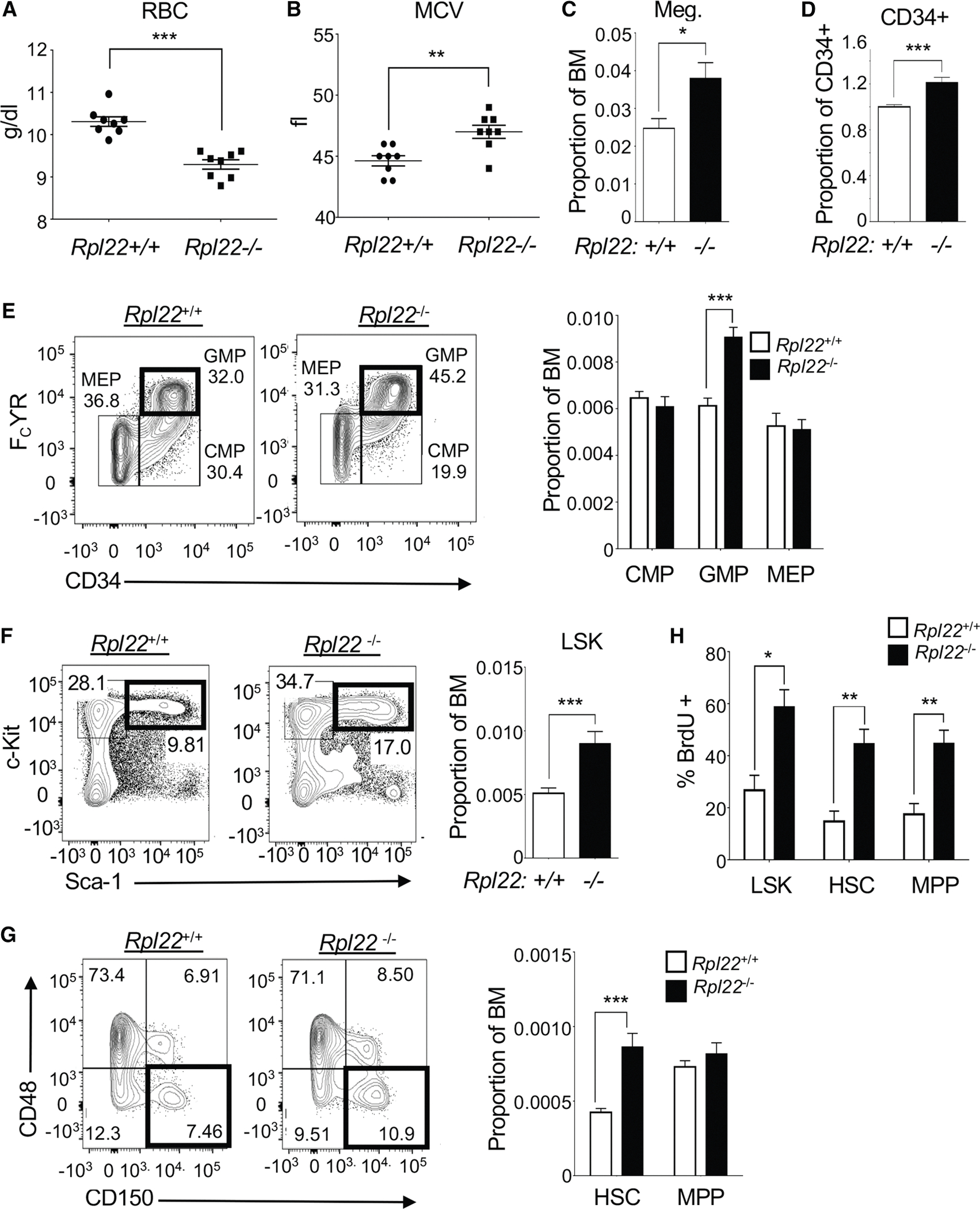
Rpl22-deficient mice exhibit MDS-like characteristics (A) Measurement of red blood cells (RBCs; g/dL) in the peripheral blood of *Rpl22*^+/+^ and *Rpl22*^−/−^ mice (*n* = 8). (B) Measurement of the mean corpuscular volume (MCV) of RBCs from *Rpl22*^+/+^ and *Rpl22*^−/−^ mice (*n* = 8). (C and D) The proportion of CD41^+^, FSC-high megakaryocytes (*n* = 9) and CD34^+^ progenitors (*n* = 18) in the bone marrow of *Rpl22*^+/+^ and *Rpl22*^−/−^ mice was measured by flow cytometry. (E) Representative histograms of lineage− cKit+ (LK) subsets, defined by FcγR and CD34, including granulocyte-macrophage progenitors (GMPs; LK/CD34+/FcγR high), in the bone marrow of *Rpl22*^+/+^ and *Rpl22*^−/−^ mice. Proportions of the indicated populations are represented graphically on the right ± standard error (*n* = 9). (F and G) Representative histograms of LSK and HSCs (LSK/CD150^+^/CD48^−^) in the bone marrow of *Rpl22*^+/+^ and *Rpl22*^−/−^ mice. The proportions are represented graphically as the mean ± standard error (*n* = 15). (H) Graphical representation of flow cytometric analysis of BrdU incorporation in LSK cells, HSCs, and MPPs (*n* = 4). All graphs were analyzed for significance using the Student’s *t* test unless otherwise specified. Error bars represent SEM; *p ≤ 0.05, **p ≤ 0.01, ***p ≤ 0.001.

To determine whether Rpl22 regulates MDS and AML progression, we assessed whether loss of *Rpl22* disrupted murine hematopoiesis. Similar to patients with MDS, *Rpl22*^−/−^ mice displayed a significant reduction in red blood cells (RBCs) with increased mean corpuscular volume (MCV), as is observed in macrocytic anemia associated with MDS (Figures 2A and 2B).^29^ Rpl22-deficient mice also exhibited evidence of erythroid and myeloid dysplasia (Figures S1B and S1C) and an increased frequency of megakaryocytes (Figure 2C). Finally, the bone marrow of Rpl22-deficient mice contained ~20% more CD34^+^ cells (Figure 2D) and an expansion of GMPs (Lin^−^ c-Kit^+^ CD34^+^ FCγR^high^; Figure 2E), as is typically observed in patients with higher risk for MDS and AML.^30^

Since the alterations in hematopoiesis observed in patients with MDS result from impaired function of their expanded HSC population, we sought to determine whether these same abnormalities characterized the HSCs in *Rpl22*^−/−^ mice.^31,32^ Indeed, both lineage−, Sca-1^+^, c-Kit^+^ (LSK) cells, and HSCs defined by signaling lymphocyte activation molecule (SLAM) markers (LSK/CD48^−^CD150^+^) were increased in the bone marrow of *Rpl22*^−/−^ mice relative to wild-type littermate controls (Figures 2F, 2G, S2A, and S2B).^33,34^ This expansion was associated with an increased number of proliferating cells, as HSCs and other progenitor populations in *Rpl22*^−/−^ mice exhibited greater bromodeoxyuridine (BrdU) incorporation (Figure 2H). *Rpl22*^−/−^ HSCs also exhibited a modest increase in Ki-67 staining compared to *Rpl22*^+/+^ controls (Figure S2C; *p* = 0.06).

Because inactivation of other RPs (e.g., *RPS14*) has been reported to impair hematopoiesis by activation of p53, we employed p53-deficient mice to determine whether the expansion of premalignant HSCs in *Rpl22*^−/−^ mice was p53 dependent.^13,35^ Interestingly, we found that p53 deficiency failed to suppress the expansion of LSK cells and HSCs observed in Rpl22-deficient mice (Figures S2D and S2E). Thus, as occurs in patients with MDS, Rpl22-deficient mice display an expansion of HSCs in the marrow; however, this expansion is not dependent upon p53, as it is not corrected by p53 deficiency. We previously reported that Rpl22 and its paralog Rpl22-Like1 (Rpl22l1 or Like1) antagonistically control the emergence of embryonic HSCs, with Rpl22 repressing emergence and Like1 interfering with that repression.^21^ The expansion of adult HSPCs in Rpl22-deficient mice is also dependent upon Like1, since ablation of one allele of *Rpl22l1* was sufficient to markedly reduce the number of both LSK cells and HSCs in adult bone marrow (Figures S2F–S2H).^36,37^

### Rpl22 loss impairs HSC function

**Figure 3.**
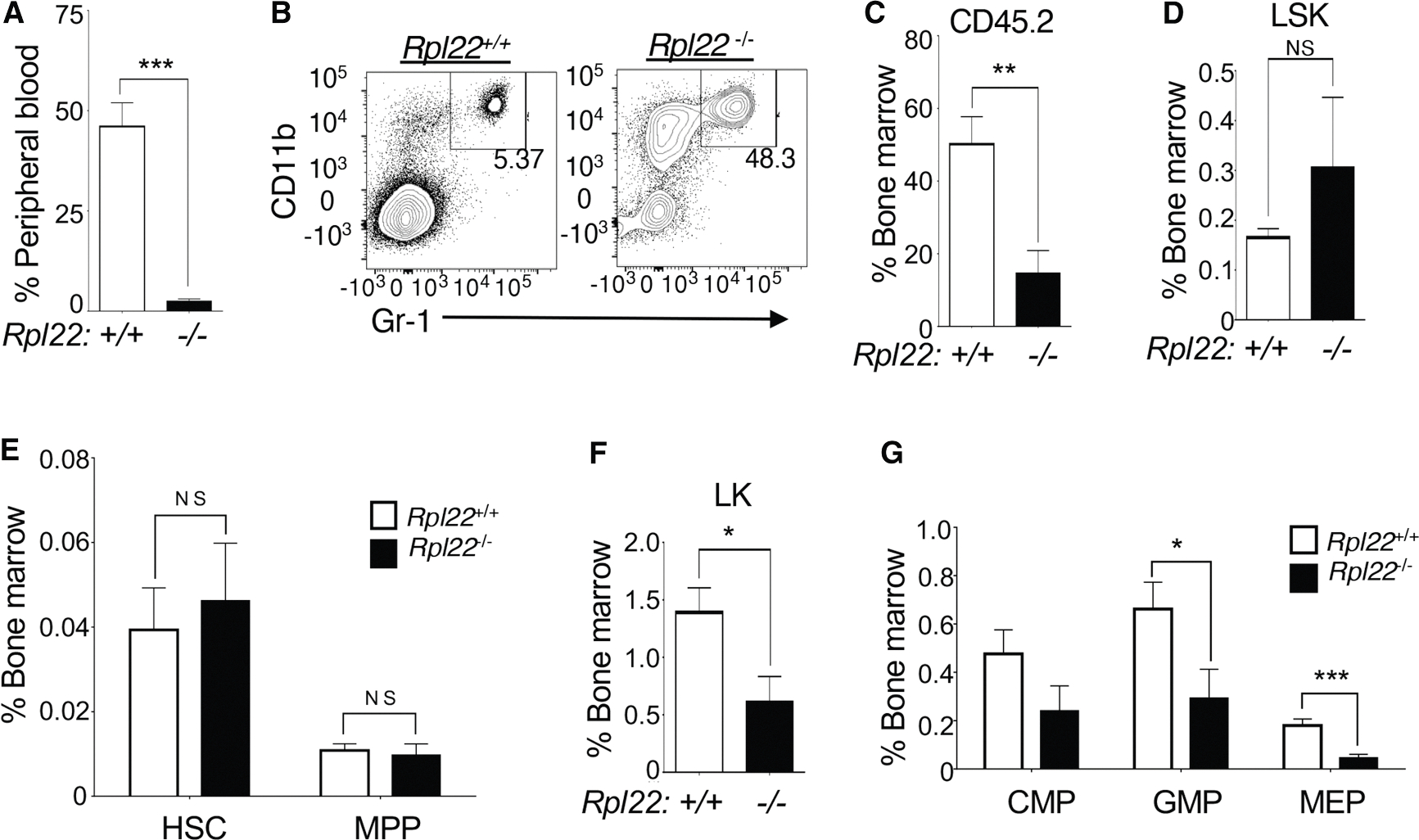
HSCs from Rpl22-deficient mice display altered stem cell function Competitive transplantation of 300 CD45.2 HSCs with 200,000 CD45.1 competitor WBM cells. All data shown represent analysis at 20 weeks post transplantation (*n* = 10). (A) Contribution of CD45.2 *Rpl22*^+/+^ and *Rpl22*^−/−^ HSCs to the peripheral blood following competitive transplantation. (B) Representative histogram of myeloid lineage cells in the peripheral blood of transplanted mice. (C–G) Contribution of CD45.2 *Rpl22*^+/+^ and *Rpl22*^−/−^ HSCs to the following bone marrow populations after competitive transplantation: (C) total bone marrow cellularity; (D) LSK; (E) HSCs and MPPs; (F) LK; and (G) LK subsets, CMP, GMP, and MEP. All graphs were analyzed for significance using the Student’s *t* test. Error bars represent standard error of the mean (SEM). *p ≤ 0.05, **p ≤ 0.01, ***p ≤ 0.001.

The alterations in hematopoiesis observed in patients with MDS have previously been shown to result from dysfunctional HSCs.^31,32,38^ To determine whether this was also true of the altered hematopoiesis observed in Rpl22-deficient mice, we performed competitive bone marrow transplantation assays using allotype-marked (CD45.2) *Rpl22*^−/−^ or *Rpl22*^+/+^ HSCs combined with whole bone marrow competitor (WBM; CD45.1). Analysis of peripheral blood 20 weeks after transplantation revealed that *Rpl22*^−/−^ HSCs were significantly less capable of reconstituting hematopoiesis than *Rpl22*^+/+^ HSCs, as *Rpl22*^−/−^ HSCs produced very few peripheral blood leukocytes (Figure 3A). Moreover, the few leukocytes that were produced by Rpl22-deficient HSCs were profoundly biased toward the myeloid fate (CD11b^+^Gr1^+^; Figure 3B), consistent with the basal myeloid-bias observed in the donor *Rpl22*^−/−^ mice and in patients with MDS.^39^

The failure of *Rpl22*^−/−^ HSCs to reconstitute hematopoiesis could have resulted from their failure to engraft or, alternatively, from failure to produce mature hematopoietic cell lineages following engraftment. To evaluate the extent of engraftment by Rpl22-deficient HSCs, we assessed donor chimerism in the bone marrow of transplanted mice. While *Rpl22*^−/−^ HSCs contributed less to overall bone marrow cellularity than did *Rpl22*^+/+^ HSCs (Figure 3C), the contribution of *Rpl22*^−/−^ HSCs to the recipient bone marrow was nearly two times (1.8-fold) greater than to peripheral blood (Figure S2I). Together these data suggest that the MDS-like phenotype observed in *Rpl22*^−/−^ mice results from HSC dysfunction, where Rpl22-deficient HSCs are capable of self-renewal, as shown by their ability to engraft and expand in the bone marrow, but are impaired in their ability to produce lineage-committed downstream progenitors or peripheral leukocytes.

To identify the stages of hematopoiesis impaired by Rpl22 deficiency, we assessed the contribution of *Rpl22*^−/−^ HSCs to hematopoietic progenitor populations relative to that of *Rpl22*^+/+^ HSCs. Interestingly, transplanted *Rpl22*^−/−^ HSCs produced LSK cells, HSCs, and multipotent progenitors (MPPs, LSK/CD150^−^CD48^−^) as effectively as *Rpl22*^+/+^ HSCs (Figures 3D, 3E, and S2J). However, development of *Rpl22*^−/−^ progenitors beyond the MPP stage was more severely impaired, as LK cells, and all of their subsets (GMP; LK/CD34^−^FCγR^low^ megakaryocyte-erythroid progenitors [MEPs] and LK/CD34^+^FCγR^low^ common myeloid progenitors [CMPs]) were reduced relative to mice transplanted with *Rpl22*^+/+^ HSCs, with GMP and MEPs exhibiting the most profound reductions (Figures 3F and 3G). Consequently, Rpl22-deficient HSCs are capable of engraftment, expansion, and generation of MPPs but are impaired in their ability to give rise to downstream, committed progenitors.

### Rpl22 deficiency promotes the development of leukemia

**Figure 4.**
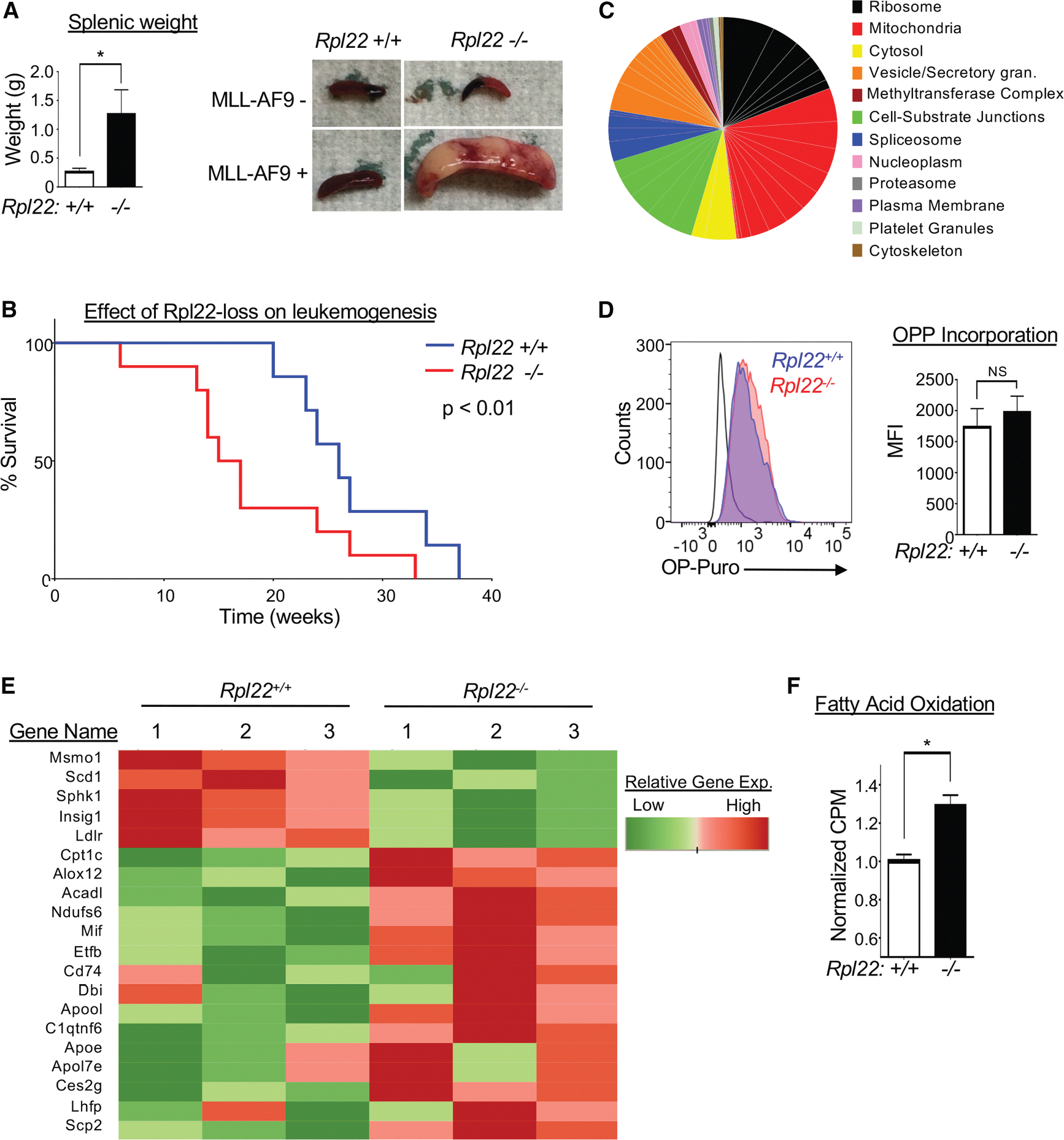
Rpl22 deficiency facilitates leukemogenesis (A) Disease burden on *MLL-AF9* knockin *Rpl22*^+/+^ and *Rpl22*^−/−^ mice was measured at 15 weeks of age (*n* = 5). Representative spleens are depicted along with a graphical representation of the mean ± SEM of splenic weights (*p* < 0.05). (B) Survival analysis of *MLL-AF9* knockin *Rpl22*^−/−^ (*n* = 10) and *Rpl22*^+/+^ (*n* = 7) mice. Significance was determined using a Mantel-Cox test. (C) Gene Ontology (GO) analysis of genes differentially expressed by *Rpl22*^−/−^ HSCs. The differentially expressed genes were identified by RNA-seq and subjected to GO analysis using the GO Cellular Components Database. Signficant gene sets were categorized and represented as a pie chart with the size of the pie wedges reflecting EnrichR combined score for a given GO category. (D) Protein synthesis was measured in HSC by assessing the amount of O-propargyl-puromycin (OPP) incorporated into nascent polypeptides by flow cytometry after exposure to OPP for 1h. A representative FACS histogram of OPP incorpration by HSC is depicted and expressed graphically as the mean +/− SEM of the mean fluorescence intensity (MFI). (E) Heatmap depicting the expression levels in HSCs of genes involved in fatty acid metabolism (*n* = 3). (F) Measurement of fatty acid oxidation (FAO) using C14-labeled palmitate in *Rpl22*^+/+^ and *Rpl22*^−/−^ LSK cells. FAO activity is depicted graphically as the mean ± SEM of triplicate measurements. All graphs were analyzed for significance using the Student’s *t* test. *p ≤ 0.05.

Since self-renewal without the capacity to differentiate characterizes a premalignant state, we reasoned that *Rpl22*^−/−^ HSCs should be predisposed to leukemic transformation.^40^ Consequently, we assessed whether *Rpl22*^−/−^ HSCs were predisposed to transformation using the MLL-AF9 oncogene knockin model of AML.^41,42^ Indeed, MLL-AF9 knockin mice exhibited splenomegaly comprising Mac1^+^ leukemic cells (Figure S3A), with the *Rpl22*^−/−^ mice exhibiting a far greater leukemic burden at 15 weeks of age, as shown by splenic weight (Figure 4A). Furthermore, Rpl22-deficient MLL-AF9 knockin mice developed and succumbed to leukemia much more rapidly than their *Rpl22*^+/+^ counterparts (Figure 4B).

### Rpl22 deficiency alters the metabolism of HSCs and resulting leukemias

To determine how Rpl22 deficiency impairs the function of HSCs and enhances their transformation potential, we performed whole-transcriptome analysis on HSCs from *Rpl22*^+/+^ and *Rpl22*^−/−^ mice. From this analysis, we found that Rpl22 deficiency altered the expression of more than 500 genes (Figure S3B; Table S1). Gene Ontology (GO) analysis using the Cellular Components Database revealed that Rpl22 deficiency most significantly affects the expression of clusters of genes associated with the ribosome and mitochondria (Figure 4C; Table S2). Because HSC function has been reported to be impaired by reductions in protein synthesis, and because RP mutations are linked to significant decreases in global protein synthesis, we sought to determine whether Rpl22 deficiency was impairing HSC function by attenuating protein synthesis.^8,43,44^ To test this possibility, we measured protein synthesis in HSCs *in situ* by monitoring O-propargyl-puromycin (OPP) incorporation into nascent polypeptides using flow cytometry.^43,44^ Surprisingly, we observed that the incorporation of OPP into newly synthesized proteins by *Rpl22*^−/−^ HSCs (red) was equivalent to that incorporated by *Rpl22*^+/+^ HSCs (blue) (Figure 4D). Therefore, in contrast to what has been observed for HSCs upon inactivation of other RPs, this analysis revealed the unexpected finding that the alteration of HSC function caused by Rpl22 deficiency is not associated with an attenuation of global protein synthesis.^8,13,43,45^

Because global protein synthesis was not impaired by Rpl22 loss, we sought to determine whether altered mitochondrial function was contributing to the impaired function of Rpl22-deficient HSCs, since mitochondrial function, particularly oxidative phosphorylation and fatty acid oxidation (FAO), was another GO class exhibiting an altered expression signature in Rpl22-deficient HSCs (Figures 4C–4E; Table S2). To determine whether these changes in gene expression altered mitochondrial function, we performed metabolic analysis on *Rpl22*^+/+^ and *Rpl22*^−/−^ LSK cells. We observed no differences in mitochondrial biomass or in extracellular acidification rate (ECAR), a surrogate measure of glycolysis (Figures S4A and S4B). Consistent with the lack of change in ECAR, phospho-AMP activated protein kinase (pAMPK), ATP, and lactate levels were also unaffected by Rpl22 deficiency (Figure S4C). *Rpl22*^−/−^ LSK cells did exhibit a reduced oxygen consumption rate (OCR), suggesting a reduction in cellular capacity to mediate oxidative respiration (Figure S4D). The reduction in oxygen consumption is associated with reduced expression of a key component of the electron transport chain, *Atp5e*, which is required for oxygen consumption by electron transport complex (ETC) IV (Table S1).^46^ Interestingly, the expression of several genes involved in fatty acid metabolism was increased in Rpl22-deficient HSCs (Figure 4E). These alterations in gene expression are noteworthy because increased dependence on FAO has been implicated in HSC self-renewal, leukemic stem cell function, and cancer stem cell function.^47–49^ Consistent with the increased expression of genes involved in lipid oxidation, FAO was increased in *Rpl22*^−/−^ LSK cells (Figure 4F). It should be noted that attenuating FAO using the FAO inhibitor etomoxir^50,51^ did not restore oxygen consumption in *Rpl22*^−/−^ HSPCs (Figure S4D).

**Figure 5.**
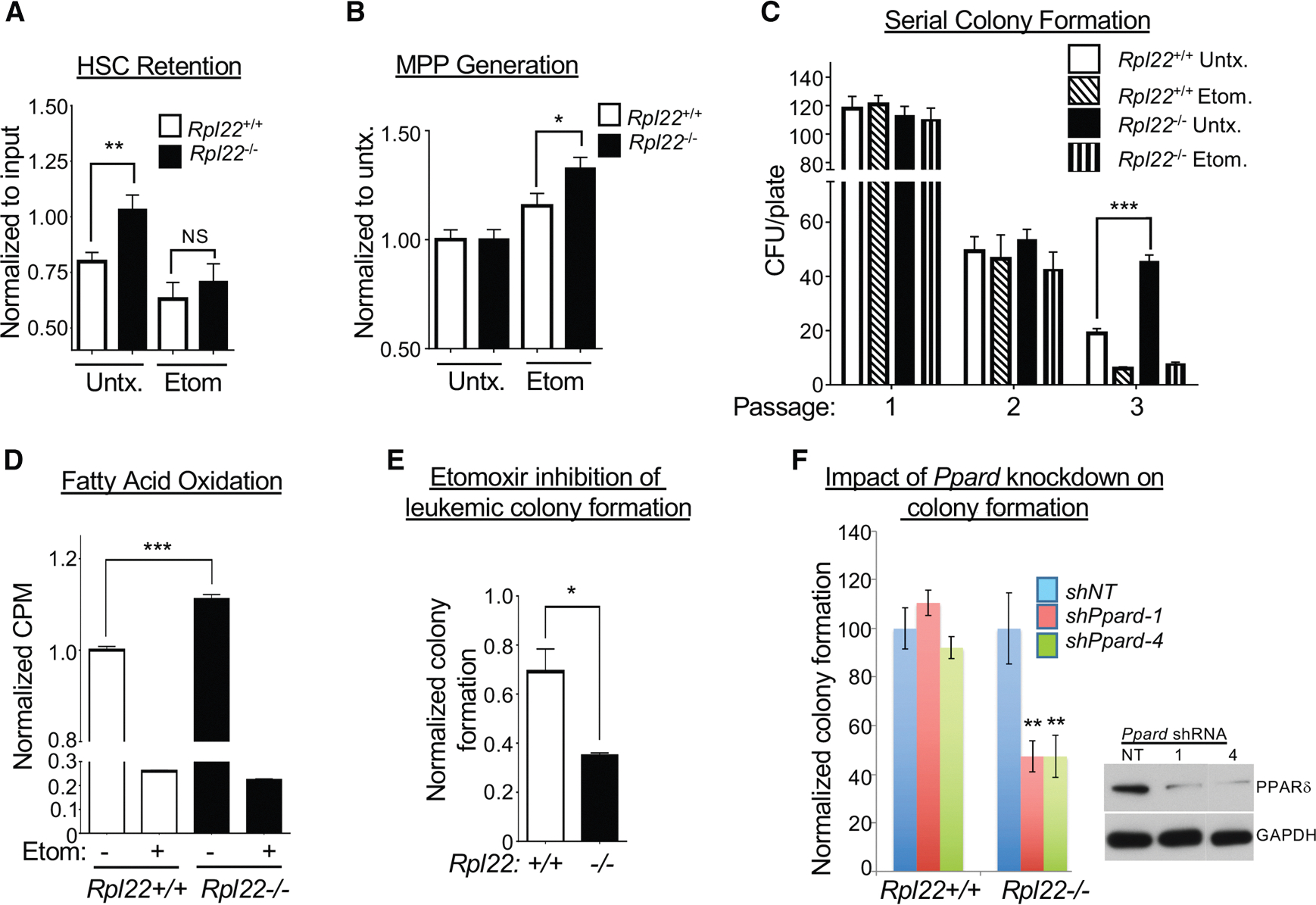
The enhanced retention of progenitor activity by Rpl22-deficient HSPCs depends on FAO (A and B) Flow cytometric assessment of the retention of the HSC immunophenotype (CD150^+^CD48^−^) or differentiation into MPPs following 2 days of culture in methylcellulose in the presence or absence of 200 μM etomoxir (*n* = 9). (C) Serial colony-forming ability of *Rpl22*^+/+^ and *Rpl22*^−/−^ HSPCs treated with vehicle (DMSO) or etomoxir. Mean ± SEM of colonies of triplicate cultures serially passaged three times is depicted graphically. (D) Measurement of FAO using C14-labeled palmitate in MLL-AF9-transformed LSK from *Rpl22*^+/+^ and *Rpl22*^−/−^ mice. Triplicate measurements of FAO activity are depicted graphically as the mean ± SEM. (E) Effect of etomoxir treatment on colony formation by MLL-AF9-transformed LSK cells from *Rpl22*^+/+^ and *Rpl22*^−/−^ mice. Triplicate measurements of colony formation in methylcellulose are depicted graphically as the mean ± SEM. (F) PPARδ loss selectively attenuates colony formation by *Rpl22*^−/−^ leukemias. MLL-AF9-transformed leukemias from *Rpl22*^+/+^ and *Rpl22*^−/−^ mice were transduced with shRNA targeting *Ppard*, following which their colony-forming capacity in methylcellulose was assessed. The mean ± SEM of triplicate measurements is depicted graphically, and the effect of the shRNA on PPARδ expression was assessed on B16 melanoma cells by immunoblotting. All graphs were analyzed for significance using the Student’s *t* test. Error bars represent standard error of the mean (SEM). *p ≤ 0.05, **p ≤ 0.01, ***p ≤ 0.001.

Since increased FAO has been implicated in the self-renewal of HSCs, we asked whether explanted Rpl22^−/−^ HSCs exhibited a prolonged retention of their SLAM-marked HSC phenotype (LSK, CD150^+^, CD48^−^) upon culture *in vitro*.^47^ Indeed, although a significant proportion of *Rpl22*^+/+^ HSCs lost their HSC phenotype after 2 days in culture, it was fully retained by *Rpl22*^−/−^ HSCs (Figure 5A). Moreover, retention of the HSC phenotype by *Rpl22*^−/−^ HSCs was dependent on FAO, as it was abrogated by inhibition of FAO using etomoxir (Figure 5A).^50,51^ Blockade of FAO was not cytotoxic to *Rpl22*^−/−^ HSCs but instead induced their differentiation into MPPs (Figure 5B). *Rpl22*^−/−^ HSPCs also exhibited enhanced function, as they retained a greater capacity to form colonies in methylcellulose following serial passage (Figure 5C). This capability was also attenuated by pharmacologic inhibition of FAO (Figures 5C and S4E). Together, these findings suggest that the increased self-renewal and inability of *Rpl22*^−/−^ HSCs to support the development of downstream committed progeny results from an alteration in cellular metabolism. Specifically, *Rpl22*^−/−^ HSCs exhibit enhanced FAO, which promotes self-renewal.^47^

Since increased FAO was required for the enhanced self-renewal exhibited by *Rpl22*^−/−^ HSCs, we next asked whether enhanced FAO also contributed to increased leukemic potential of the Rpl22-deficient MLL-AF9 leukemia cells. We found that, similar to non-transformed HSCs, MLL-AF9-transformed LSK cells from *Rpl22*^−/−^ mice exhibited increased FAO (Figure 5D). The *Rpl22*^−/−^ leukemia did not exhibit the reduction in oxygen consumption observed in primary Rpl22-deficient LSK cells, suggesting that this defect is not retained during transformation (Figure S4F). The increase in FAO appears to play an important role in maintaining the leukemic potential of *Rpl22*^−/−^ leukemia cells, since treatment with the FAO inhibitor etomoxir was far more effective in inhibiting colony formation by *Rpl22*^−/−^ leukemia cells than by their *Rpl22*^+/+^ counterparts (Figure 5E). Moreover, the ability of Rpl22-deficient leukemias to form colonies was preferentially dependent upon the transcription factor PPARδ, a master regulator of FAO that has been implicated in the ability of FAO to promote HSC renewal (Figure 5F).^47^ Indeed, short hairpin RNA (shRNA) knockdown of *Ppard* selectively attenuated colony formation by *Rpl22*^−/−^ leukemias (Figure 5F), indicating that Rpl22 loss promotes leukemic colony formation by upregulating FAO in a PPARδ-dependent manner.

### Rpl22 regulation of FAO and leukemia pathogenesis

**Figure 6.**
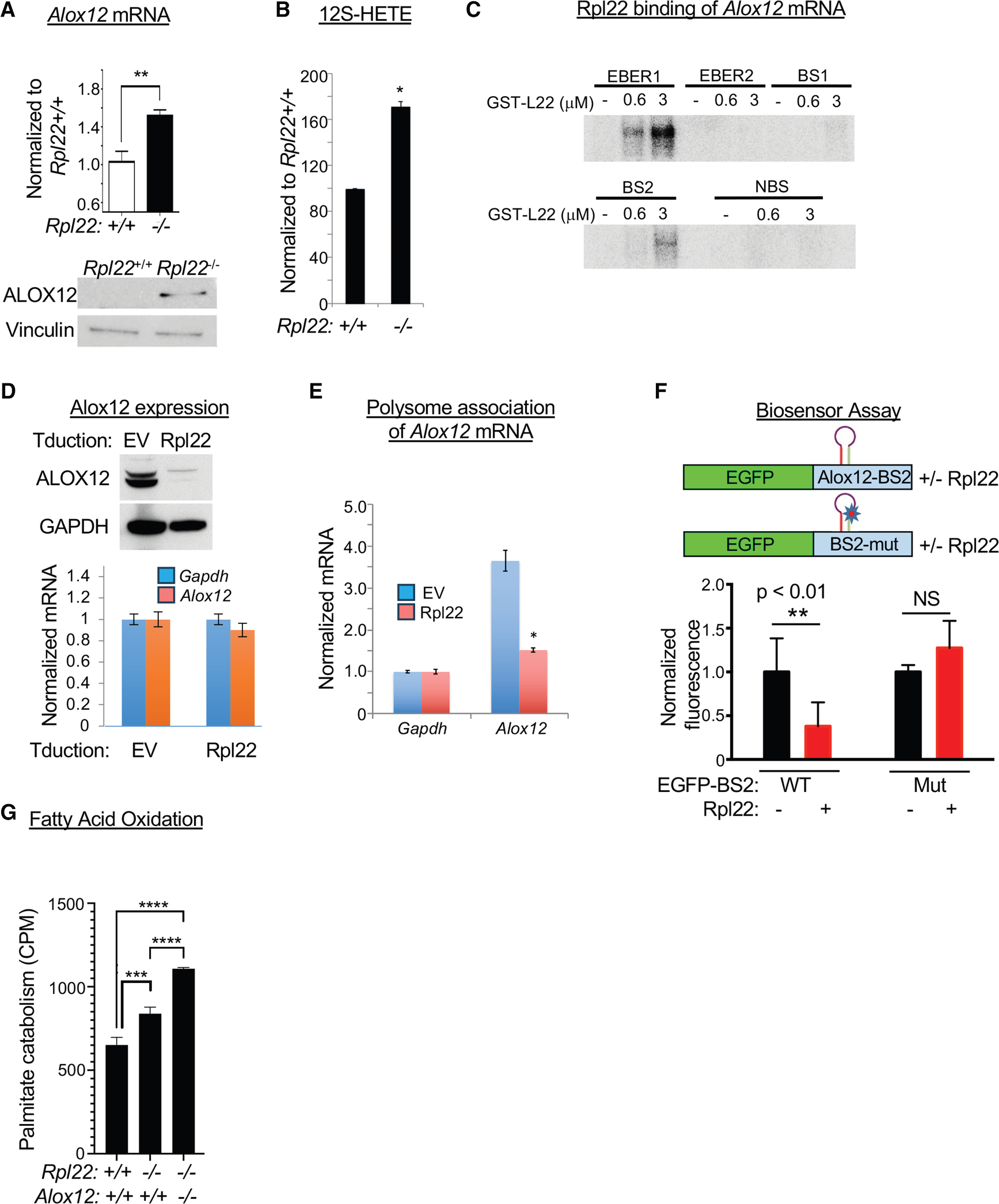
Alox12 is an Rpl22 target but is not responsible for elevated lipid metabolism (A) qPCR and immunoblot analysis of *Alox12* mRNA and protein expression, respectively, in *Rpl22*^+/+^ and *Rpl22*^−/−^ HSCs and LSK cells. Triplicate RT-qPCR measurements are represented graphically as mean ± SEM, with the expression level in *Rpl22*^−/−^ HSCs normalized to that in *Rpl22*^+/+^ HSCs (*n* = 3). Vinculin was used as an immunoblotting loading control. (B) The levels of 12S-HETE in *Rpl22*^+/+^ and *Rpl22*^−/−^ LSK cells were measured in triplicate by ELISA. The mean level ± SEM was normalized to that in *Rpl22*^+/+^ LSK cells and depicted graphically. (C) Assessment of GST-Rpl22 binding to the indicated fragments of *Alox12* mRNA (BS1, binding site 1; BS2, binding site 2; and NBS, non-binding site) as measured by EMSA analysis. *EBER1* and *EBER2* RNA served as positive and negative controls, respectively. (D) Repression of Alox12 expression by Rpl22. *Rpl22*^−/−^ MEFs ectopically expressing the coding region of murine *Alox12* were transduced with either the empty vector (EV) control or pMiG-Rpl22, following which the effect on Alox12 expression was assessed by immunoblotting and RT-qPCR as in (A). (E) Control of *Alox12* mRNA inclusion in polysomes. Cells from (D) were fractionated on a continuous sucrose gradient, which separated cellular RNA into polysome-associated and free fractions. The amount of free and polysome-associated *Alox12* and *Gapdh* RNA was quantified by RT-qPCR and normalized to that in the EV-transduced control, as in (D). (F) Transfer of Rpl22 responsiveness to mRNA encoding GFP using the Rpl22-binding site in *Alox12* mRNA. *Gfp* constructs appended in frame with intact or mutated *Alox12* BS2 were injected into zebrafish embryos at the one-cell stage alone or with mRNA encoding Rpl22 (Figure S5G). After 10 h, GFP fluorescence was quantified and depicted graphically after normalization to the fluorescence intensity of the intact BS2 construct in control-injected fish. All data are representative of three experiments performed unless otherwise stated. Significance for pairwise tests was assessed using the Student’s *t* test while multiple comparisons were assessed by one-way ANOVA. Error bars represent standard error of the mean (SEM). *p ≤ 0.05, **p ≤ 0.01, ***p ≤ 0.001, ****p ≤ 0.0001. (G) Measurement of FAO using C14-labeled palmitate on LSK cells from mice of the indicated genotypes. Triplicate measurements of FAO activity are depicted graphically as the mean ± SD. ***p < 0.0004; ****p < 0.0001.

Because Rpl22 is an RNA-binding protein, we hypothesized that Rpl22 might control FAO in HSCs by modulating the activity of mRNA targets encoding regulators of this process. Using M-fold to predict RNA secondary structure, we found that several of the transcripts encoding regulators of FAO bear the consensus stem-loop structure recognized by Rpl22 (Figures S5A–S5E). Among these differentially expressed targets (Figure 4E), arachidonate lipoxygenase-12 (Alox12) was of particular interest, both because there were two consensus Rpl22-binding sites in the coding regions of both mouse *Alox12* and human *ALOX12* (Figures S5B and S5F) and because Alox12 converts polyunsaturated fatty acids to the activating ligands for a master transcriptional regulator of FAO, PPARδ.^52,53^ Analysis of Alox12 protein and mRNA levels revealed that the increase in Alox12 protein levels exceeded that of its mRNA (Figure 6A), suggesting post-transcriptional regulation. The increase in Alox12 expression was accompanied by increased generation of the arachidonic acid breakdown product it generates, 12S-HETE (Figure 6B). We next wished to determine how the expression of Alox12 was controlled by Rpl22. To determine whether Rpl22 bound to the two consensus binding sites in the coding sequence of *Alox12* mRNA, we performed electrophoretic-mobility shift analysis (EMSA) (Figure 6C). EMSA analysis confirmed that Rpl22 bound strongly to positive-control RNA, EBER1, but not negative-control EBER2.^54^ Moreover, Rpl22 also bound to the second predicted binding site (BS2) of *Alox12* but not to the first predicted binding site (BS1) or to NBS, an RNA sequence between BS1 and BS2 that lacks the Rpl22 target motif (Figure 6C). In addition to its ability to bind to *Alox12* mRNA, Rpl22 is capable of post-transcriptionally repressing the expression of Alox12 upon ectopic expression in Rpl22-deficient mouse embryonic fibroblasts (MEFs) (Figure 6D). Rpl22 appears to be regulating Alox12 by controlling its inclusion in actively translating polysomes, since Rpl22 reintroduction into Rpl22-deficient MEFs displaces *Alox12* mRNA for heavy polysomes (Figure 6E). Moreover, appending the Rpl22-binding site in *Alox12* mRNA (BS2) to a heterologous mRNA (GFP) conferred responsiveness to Rpl22 regulation (Figures 6F and S5G), collectively indicating that Rpl22 controls HSC function by directly binding *Alox12* mRNA and regulating Alox12 protein expression. To assess whether Alox12 is the critical link between Rpl22 deficiency and elevated FAO, we crossed *Rpl22*^−/−^ mice to Alox12 deficiency (*Rpl22*^−/−^*Alox12*^−/−^)^55^ and assessed FAO in HSPCs from these mice using C^14^-palmitate catabolism (Figure 6G). While *Rpl22*^−/−^ HSPCs clearly exhibited elevated FAO activity as measured by C^14^-palmitate catabolism, indicating that Rpl22 loss elevates FAO, FAO was not attenuated by the genetic ablation of *Alox12* (Figure 6G). Together, these data demonstrate that, despite Alox12 elevation and the increased presence of its 12S-HETE product, the Rpl22 target Alox12 is not responsible for the enhanced FAO observed in *Rpl22*^−/−^ HSPCs.

### Role of Lin28b in promoting the pathogenesis of Rpl22^−/−^ leukemias

**Figure 7.**
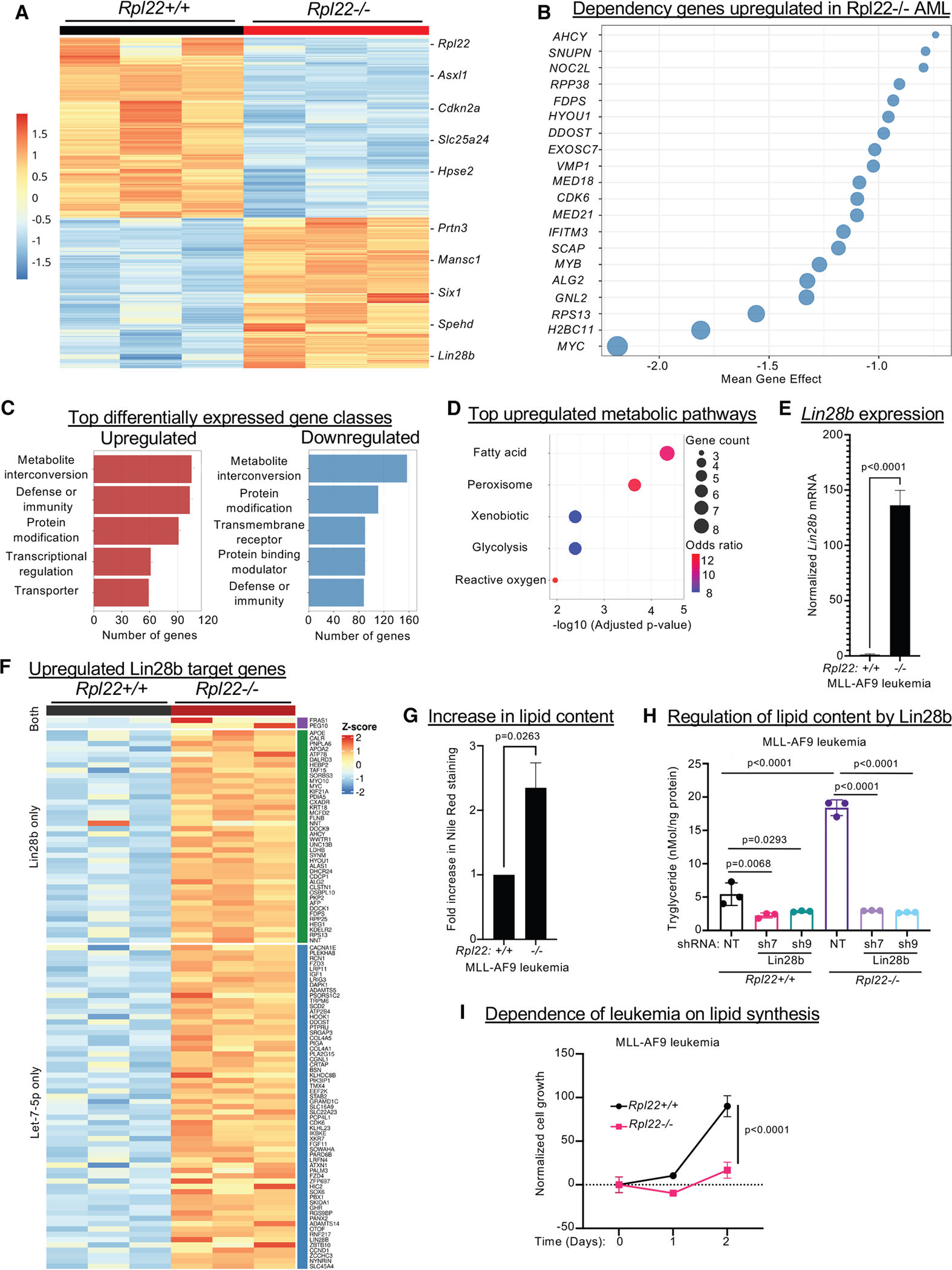
Rpl22-deficient leukemia cells are dependent on elevated lipid synthesis (A) Leukemia explants from MLL-AF9-knockin *Rpl22*^+/+^ (M82) and *Rpl22*^−/−^ (M109) mice were analyzed by RNA-seq. A heatmap of 2,671 differentially expressed genes is displayed. (B) Top 20 upregulated essential genes in *Rpl22*^−/−^ leukemias identified using the CRISPR DEPMAP CERES dataset across human leukemia cell lines and displayed as a bubble plot of the impact of their genetic disruption on leukemia survival. (C) Differentially expressed genes were subjected to pathway analysis (Protein Analysis Through Evolutionalary Relationships; PANTHER) with the top upregulated and downregulated pathways displayed. The number of differentially expressed genes per pathway is indicated. </p/>(D) Bubble plot of the top five Reactome pathways depicting the most upregulated metabolic pathways in *Rpl22*^−/−^ MLL-AF9 leukemias. (E) qPCR analysis of *Lin28b* mRNA expression in *Rpl22*^+/+^ and *Rpl22*^−/−^ MLL-AF9 leukemias. Statistical significance was calculated using a two-tailed *t* test with Welch’s correction. Normalized Lin28b expression is depicted graphically as the mean +/− SD. (F) Heatmap of 103 Lin28b gene targets induced in *Rpl22*^−/−^ MLL-AF9 leukemias, subdivided based on whether they are direct Lin28b targets or are indirectly regulated through Lin28b modulation of Let7 micro-RNAs (mIRs). (G) Lipid content of *Rpl22*^+/+^ and *Rpl22*^−/−^ leukemias as measured by Nile red staining. Statistical significance was calculated using a two-tailed *t* test with Welch’s correction. Normalized lipid content is depicted graphically as mean +/− SD. (H) Triacylglycerol (TG) levels were measured on detergent extracts of *Rpl22*^+/+^ and *Rpl22*^−/−^ leukemias using a colorimetric assay quantifying oxidized glycerol liberated by lipase digestion. Mean ± SD of triplicate measures of nMol TG per ng of protein were expressed graphically. Statistical significance was determined using one-way ANOVA. (I) The effect of inhibiting TG synthesis using DGAT1 inhibitor (DGAT1-IN-1) at 25 μM on the growth of *Rpl22*^+/+^ and *Rpl22*^−/−^ leukemias was assessed by 3-(4,5 dimethylthiazol-2-yl)-2,5-diphenyltetrazoliumbromide (MTT) assay. Triplicate measures were expressed graphically as mean ± SD for each different drug concentration. Statistical significance was determined by two-way ANOVA.

To gain insight into the molecular basis for the control of lipid metabolism by Rpl22 and to make a broader assessment of the regulation of leukemogenesis by Rpl22, we performed RNA sequencing (RNA-seq) on *Rpl22+/+* and *Rpl22*^−/−^ leukemias that developed in the MLL-AF9 transgenic mice (Figures 7A and S6A). The analysis revealed 2,671 differentially expressed genes, with 1,219 being induced and 1,452 being repressed in *Rpl22*^−/−^ leukemias (Figure 7A; Table S3). Genes upregulated in *Rpl22*^−/−^ leukemias include many that have previously been implicated in AML pathogenesis (Figure 7A). *Prtn3* and *Laptm4b* are reported to promote leukemogenesis by regulating STAT3 signaling, while *Dock1* is a guanine nucleotide exchange factor thought to promote leukemogenesis through Notch activation.^56–59^ Key transcriptional regulators are also induced in *Rpl22*^−/−^ leukemias including *Mansc1* (MN1), which serves as a critical transcriptional co-factor in MLL-rearranged leukemias, and *Six1*, which promotes leukemia by regulating the expression of glycolytic genes (Figure 7A)^60,61^. Also among the induced genes are critical metabolic regulators of leukemia progression (Table S3). *Cyb561* promotes leukemogenesis through reactive oxygen species (ROS) induction and long non-coding RNA (lncRNA) *Spehd* regulates oxidative phosphorylation and mitochondrial membrane potential required for stem cell function.^62,63^ Many of the induced genes have also been identified as essential for leukemia survival in CRISPR screens (Figure 7B). Finally, the repressed genes in *Rpl22*^−/−^ leukemias are similarly implicated in leukemia pathogenesis including *Asxl2*, *Cdkn2a*, and *Hpse2*, which have been found to be frequently mutated in leukemias (Figues 7A and S6B).^64–67^ Among the diverse differentially regulated genes in *Rpl22*^−/−^ leukemias, 10% are linked to metabolism, with fatty acid metabolism being the most enriched, upregulated metabolic pathway (Figures 7C, 7D, and S6C–S6E). Together, these data indicate that Rpl22 regulates leukemic progression by broadly regulating the expression of critical regulators falling in numerous GO groups but particularly in lipid metabolism.

Lin28b has been implicated as a key regulator of both leukemia pathogenesis and lipid metabolism, raising the possibility that Lin28b induction may play a key role in regulating lipid metabolism in *Rpl22*^−/−^ leukemias.^68–75^ We have previously shown that Rpl22 loss promotes development of T acute lymphoblastic leukemia (T-ALL) through Lin28b induction.^22^ *Lin28b* is also one of the most upregulated genes in leukemias that arose in the MLL-AF9 Tg *Rpl22*^−/−^ mice (Figures 7A–7E). Importantly, of the 1,219 genes upregulated in Rpl22-deficient leukemias, nearly 10% (102) are either direct Lin28b targets or indirect targets regulated through Lin28b effects on Let-7 micro-RNAs (mIRs) (Figure 7F; Table S4).^76,77^ These targets include many that are required for leukemia survival, such as Myc, Rps13, and CDK6 (Figure S7A) as well as those implicated in lipid metabolism (Figure S7B). Along with the expression signature, Nile red staining revealed that *Rpl22*^−/−^ leukemias displayed greater lipid content (Figure 7G). To determine whether the increased lipid content resulted from Lin28b induction, we knocked Lin28b down using shRNA (Figure S7C). Indeed, Lin28b knockdown reduced triacylglycerol (TG) synthesis preferentially in *Rpl22*^−/−^ leukemias (Figure 7H). Finally, inhibition of acyl-coenzyme A (CoA):diacylglycerol acyltransferase 1 (DAGAT1),^78^ a critical enzyme in TG synthesis,^79^ resulted in preferential attenuation of the survival of *Rpl22*^−/−^ leukemias (Figure 7I), indicating that Lin28b-mediated promotion of lipid synthesis is critical for pathogenesis of *Rpl22*^−/−^ leukemias.

## DISCUSSION

In this report, we provide evidence that RP insufficiency alters HSC function and increases the predisposition to leukemia through a novel mechanism that does not involve attenuation of global protein synthesis. Instead, HSC function is altered because of the dysregulated expression of selected Rpl22 gene targets that impact metabolism. *RPL22* expression is reduced in human MDS and AML, including at the stem cell level, and is associated with reduced survival. The link between reduced *RPL22* expression and poor survival is also observed in a mouse leukemia model lacking Rpl22. As indicated, the mechanistic link between Rpl22 loss and enhanced leukemogenic potential is distinct from that of other cases of RP insufficiency in that it does not involve attenuation of global protein synthesis.^8,13,43^ Instead, *Rpl22* inactivation creates a premalignant state characterized by enhanced HSC self-renewal and impaired generation of downstream committed progenitors by altering metabolism. Specifically, Rpl22 loss decreases oxygen consumption and increases the dependence of HSCs and leukemias on FAO. These observations are consistent with previous reports demonstrating that enhanced FAO promotes HSC self-renewal and cancer stem cell activity, including leukemia stem cells.^47–49,80,81^ The increased FAO is associated with induction of a regulator of FAO that is a direct Rpl22 target, Alox12,^52,82^ but Alox12 is not responsible for increased FAO since its loss does not return FAO to baseline. Instead, we identified induction of the stemness factor Lin28b in the resulting leukemias, which is a key driver of the enhanced lipid synthesis (TG) that supports Rpl22-deficient leukemia survival. Together, these observations indicate that Rpl22 controls HSC function and transformation potential through effects on metabolism.

Mutations in RP have previously been reported to perturb hematopoiesis and predispose to transformation^2,3,13,83–89^; however, Rpl22 deficiency appears to regulate hematopoiesis and predispose to transformation in a manner that is fundamentally different from that of other RP mutations, since Rpl22 is not essential for life and Rpl22 deficiency does not result in detectable alterations in ribosome function.^19^ Nevertheless, Rpl22 deficiency does disrupt normal hematopoiesis at multiple stages, including the perturbation of HSC function reported here.^19–22,37,90–93^ Two modes of action have been proposed to explain how insufficiency of an RNA-binding RP might regulate hematopoiesis: through effects on the ribosome itself or through extra-ribosomal activity.^9,11,94–96^ This remains a controversial issue, as there is evidence in support of both modes of action. Effects on the ribosome itself can impact HSC function by altering the overall protein synthesis rate, since normal HSC function is critically dependent on maintaining the rate of protein synthesis within a very narrow range.^43,97,98^ Rpl22 deficiency does not reduce the global protein synthesis rate, suggesting that Rpl22 deficiency does not disrupt HSC function by changing global protein synthesis. Another way RP insufficiency could affect the ribosome is that RP insufficiency may produce ribosomes with altered protein composition (specialized ribosomes), which exhibit distinct capacities to translate mRNA species bearing specific primary sequence motifs or secondary structural features.^3,12,99,100^ While evidence supporting this perspective has been reported,^92^ an opposing model suggests that RP insufficiency attenuates the translation of a selected class of mRNA species by decreasing the number of available ribosomes, rather than by altering their composition.^8^ The completely distinct mode by which RP can regulate processes is by leaving the ribosome and functioning in a physically separated manner, referred to as “extra-ribosomal” function.^96^ Clear support for this model has also been reported, including the well-documented ability of Rpl13a to dissociate from the ribosome following interferon signaling and to bind to selected mRNA targets, thereby regulating their translation.^101^ Our analysis does not definitively distinguish the mode of action through which Rpl22 functions; however, our evidence aligns best with the extra-ribosomal model. Indeed, Rpl22 assembles into the ribosome as a monomer with a single RNA-binding face,^102,103^ which is bound to the 28S rRNA, precluding it from simultaneously having direct interaction with another RNA target. Consequently, direct interaction of Rpl22 with mRNA targets, such as *Alox12*, is only possible when the RNA-binding helices of Rpl22 are free from the 28S rRNA, as is the case for the Rpl22 pool that is physically separated from the ribosome.

We have identified a number of targets through which Rpl22 regulates development, including hematopoiesis.^21,37^ We previously reported that the antagonistic balance between Rpl22 and its highly homologous paralog, Rpl22-Like1 (Rpl22l1 or Like1), controls the emergence of embryonic HSCs by directly binding and controlling the translation of *Smad1* mRNA.^21^ Rpl22 regulation of the behavior of adult stem cells is also dependent upon Like1, since Like1-insufficiency attenuates the HSC expansion observed in Rpl22-deficient mice, although this presumably does not involve effects on Smad1 expression, since Smad1 is dispensable in adult HSCs.^104^ We determined that the Rpl22-Like1 balance plays a critical role in controlling gastrulation by binding and regulating the splicing of many pre-mRNA targets, including Smad2, an essential molecular effector of gastrulation.^37^ Rpl22 regulates traversal of pre-receptor checkpoint stages for B and T lymphocytes.^20,92^ At least in T cells, the regulation of this transition is mediated through control of the unfolded protein response (UPR) or endoplasmic reticulum (ER) stress signaling.^92^ The lineage-restricted requirement for Rpl22 in regulating ER stress signaling appears to be limited to cells experiencing unusually abrupt transitions from quiescence to rapid proliferation, as is observed at the pre-receptor checkpoints.^105^ This does not appear to be relevant in cells that undergo less abrupt proliferative transitions, such as γδ T cell progenitors or adult HSCs.^19^ We report here that dysregulation of FAO by Rpl22 loss results in HSC dysfunction and is required for the survival of Rpl22-deficient leukemias, which is impaired by knockdown of the master regulator of FAO, PPARδ.^47^ Rpl22 directly binds and regulates the translation of mRNA encoding Alox12, which has been implicated in FAO^52,53^; however, Alox12 is not responsible for the increased FAO observed in Rpl22-deficient HSPC and so we do not think Alox12 plays a role in the predisposition to transformation displayed by Rpl22-deficient HSPCs. Instead, the upregulation of FAO may result from dysregulation of any number of other genes, including electron transfer flavoprotein-β (ETF-β), which both regulates FAO and supports AML pathogenesis.^106,107^

These and other observations underscore the critical role that alterations in lipid metabolism play in solid and hematologic malignancies, as well as in the control of HSC function.^47–49,80,108^ Nevertheless, the molecular basis by which enhanced FAO supports the function of normal HSCs and leukemia cells generally, and specifically in the context of Rpl22 loss, remains to be established. There are three potential non-mutually exclusive processes that might contribute to these altered behaviors. First, FAO is capable of generating energy through the contribution of NADH and FADH to the mitochondrial ETC to generate ATP.^109^ However, *Rpl22*^−/−^ HSPCs exhibited a reduction in OCR, a surrogate measure of aerobic respiration, suggesting that the generation of energy was unlikely to be responsible. The reduction in oxygen consumption is likely to result from reduced expression of *ATP5e*, which is important for the consumption of oxygen by ETC complex IV.^46^ This is consistent with our observation that *Atp5e* expression is no longer reduced in the *Rpl22*^−/−^ leukemias, which exhibit OCR levels equivalent to their *Rpl22*^+/+^ counterparts (data not shown). Second, FAO might enhance the survival of *Rpl22*^−/−^ HSPCs, since FAO has been reported to modulate the function of Bcl2 family members.^110^ This could explain how FAO promotes the survival of *Rpl22*^−/−^ leukemias. Finally, FAO also generates substantial quantities of acetyl-CoA, which can enter the Krebs cycle to generate citrate and contribute to NADPH-producing reactions. Alternatively, the acetyl-CoA can be released to the cytosol where it could alter cell behavior through the acetylation of both cytosolic and nuclear proteins.^111,112^ Indeed, our preliminary proteomic analysis has revealed that Rpl22-deficient leukemias exhibit profound alterations in protein acetylation (data not shown). Efforts are in progress to distinguish among these possibilities.

Our data suggest that upregulation of Lin28b, which plays a broader role in lipid metabolism, supports the pathogenesis of Rpl22-deficient MLL-AF9 transgenic AML through induction of TG synthesis. Lin28b is primarily expressed in fetal progenitors, where it serves as a master regulator of the fetal hematopoietic program, since its ectopic expression in adult progenitors is sufficient to recapitulate many aspects of fetal hematopoiesis.^70,113^ A key question is how Rpl22 deficiency results in Lin28b induction. While we have shown that Rpl22 can acutely regulate Lin28b expression,^22^ bioinformatic assessment of the *Lin28b* gene did not identify any Rpl22-binding motifs in either the exonic or intronic sequences, strongly suggesting *Lin28*b is not a direct Rpl22 target (data not shown). We have previously shown that Lin28b upregulation is critical for the pathogenesis of Rpl22− thymic lymphomas driven by transgenic expression of myristoylated-Akt2, and in this case Lin28b induction was dependent on nuclear factor (NF)-κB.^22^ NF-κB does not appear to be responsible for Lin28b induction in the Rpl22-deficient myeloid leukemia cells since the RNA-seq analysis did not identify an NF-κB signature. Myc is both a Lin28b target and a regulator of its expression,^71,114–116^ and so it remains unclear whether the increased expression of Myc in the Rpl22-deficient AML is the cause or a result of Lin28b induction. Interestingly, the expression of Lin28b is not elevated in adult Rpl22-deficient HSCs. This is perhaps not surprising because Lin28b is primarily expressed in fetal progenitors,^117^ but it does raise the possibility that the progenitor population for the resulting Rpl22-deficient MLL-AF9 transgenic leukemias may be of fetal origin. Irrespective of the gestational origin of the leukemia-initiating cell, Rpl22 deficiency cooperates with MLL-AF9 to lead to both Lin28b induction and the disabling of the recently reported tumor-suppressive activity of Lin28b in postnatal MLL-based leukemogenesis.^69^

Lin28b has been implicated in the pathogenesis of a variety of cancers, including AML-bearing MLL translocations.^68,71,74,118^ Lin28b is an RNA-binding protein that regulates processes, including carcinogenesis, either by direct binding to mRNA targets or indirectly by regulating the processing of *Let7* family mIRs.^118–120^ Rpl22-deficient leukemias exhibit increased expression of a large number of direct Lin28b targets as well as those regulated through *Let7* action, including Myc, which has been implicated in the Lin28b-mediated pathogenesis of numerous cancer types.^116,121,122^ Lin28b is also a well-known regulator of cellular metabolism^70,72,75^ and has been reported to support cancer progression by supporting *de novo* fatty acid synthesis.^73^ Lin28b does so through direct binding to and control of the translation of mRNAs encoding critical regulators of the synthesis of multiple TG species, SREBP-1, and SCAP.^73^ TG content is significant greater in *Rpl22*^−/−^ leukemias and they are more dependent on TG synthesis for survival than their *Rpl22*^+/+^ counterparts. While we did not observe induced mRNA encoding SREBP-1 in *Rpl22*^−/−^ leukemias, this is not surprising since their regulation by Lin28b is post-transcriptional. However, SCAP and SCD2, both targets of SREBP-1 and critical regulators of TG synthesis, were induced in *Rpl22*^−/−^ leukemias,^123,124^ suggesting that SREBP-1 activation is also responsible for the augmented production of TG in Rpl22-deficient leukemias. SCAP was also identified as an Rpl22-regulated gene upon which leukemia survival depends (Figure 7B).

Taken together, our observations indicate that the RP Rpl22 employs a novel mode of action to regulate the transformation potential of HSCs that does not involve altering global protein synthesis but is instead focused on the control of cellular lipid metabolism. Importantly, perturbations in lipid metabolism are increasingly understood to represent therapeutic vulnerabilities in AML^49,125–130^; however, past efforts to exploit this therapeutically in patients have been limited by liver toxicity.^131,132^ Our findings suggest that targeting TG synthesis may represent a more fruitful approach,^133–135^ particularly in those patients with *RPL22* insufficiency.

### Limitations of the study

While our study clearly implicates perturbation of lipid metabolism as the mechanism by which Rpl22 deficiency alters HSC function and promotes leukemia survival, these data are largely derived from *in vitro* assessments. Thus, it remains possible that leukemia pathogenesis *in vivo* is influenced by additional cellular pathways active in the bone marrow that are not effectively modeled *in vitro*. To conclusively test whether Rpl22 deficiency increases the leukemogenic potential of HSCs by enhancing FAO, a genetic intervention capable of returning FAO to baseline in oncogene-expressing Rpl22-deficient HSPCs *in vivo* is required, and this is not currently possible. In addition, our contention that increased triglyceride synthesis is important in supporting the survival of Rpl22-deficient leukemias is based on pharmacologic inhibition, which attenuates the survival of Rpl22-deficient leukemias *in vitro*; however, because pharmacologic agents have off-target effects, it remains possible that attenuation of a potential off-target activity also plays a role in the preferential impairment of Rpl22-deficient leukemias. Finally, the influence of Rpl22 insufficiency on HSC function and leukemogenesis was modeled in mice using Rpl22 deficiency. Patients with MDS and AML exhibit reductions in *RPL22* expression but not complete loss. Thus, it is possible that the pathways dysregulated by *RPL22* insufficiency in patients with MDS or AML may be less profoundly impacted or distinct from those perturbed in mice by Rpl22 deficiency.

### RESOURCE AVAILABILITY

#### Lead contact

Further information and requests for resources and reagents should be directed to and will be fulfilled by the lead contact, David Wiest (david.wiest@fccc.edu).

#### Materials availability

All reagents and novel mouse strains used in this study are available upon request from the lead contact, David Wiest.

#### Data and code availability

- The RNA-seq data generated in this study for *Rpl22*^+/+^ and *Rpl22*^−/−^ HSCs have been deposited in the Gene Omnibus Database (GEO) and can be accessed using GSE237505.
- Gene expression data on sorted LT-HSCs (Lin^−^, CD34^+^, CD38^−^, CD90^+^), ST-HSCs (Lin^−^, CD34^+^, CD38^−^, CD90^−^), GMPs (Lin^−^, CD34^+^, CD38^+^, CD123+, CD45RA+) from patients with AML/MDS and healthy controls have been deposited in the GEO database (GSE35008 and GSE35010).
- The RNA-seq data generated from *Rpl22*^+/+^ and *Rpl22*^−/−^ CD11b^*+*^Gr1^*+*^ MLL-AF9 leukemias have been deposited in the GEO database (GSE302046).

## STAR★METHODS

### METHOD DETAILS

#### Mice

Use of animal models was approved by the Fox Chase Cancer Center Institutional Animal Care and Use Committee. Mice were maintained in the Association for Assessment and Accreditation of Laboratory Animal Care-accredited Laboratory Animal Facility at Fox Chase Cancer Center and were handled in compliance with guidelines established by the Institutional Animal Care and Use Committees. Young adult *Rpl22+/+, Rpl22*^−/−^, and *Alox12*^−/−^ mice (8–12wks of age) were used in all experiments and had been backcrossed to the C57BL/6 background.^19,55^ CD45.1 allotype marked C57BL/6 mice were purchased from Jackson Labs (Bar Harbor, ME). *MLL-AF9* knockin mice were also purchased from Jackson Labs,^42^ backcrossed with Rpl22-deficient mice in our colony, following which survival analysis was performed on littermates. For survival analysis, *MLL-AF9* knockin mice were sacrificed upon developing symptoms of disease (i.e., difficulty breathing, hunched posture, poor grooming, or obvious splenic protuberance) or at specified times for disease burden analysis.

#### Patient database and survival data

All human samples used in this study were obtained with informed consent with approval by the Institutional Review Board of the Albert Einstein College of Medicine. The expression of ribosomal protein genes and their correlations with clinical parameters were derived from gene expression studies of 183 MDS and 17 healthy CD34^+^ control bone marrow samples (GSE19429). AML survival curve and Rpl22 expression data was obtained from the Beat AML consortium.^24^ Patient samples used in generation of the survival curve were obtained within 60 days of diagnosis. AML survival data was confirmed with 200 AML samples from TCGA database. Survival curves were calculated by Kaplan Meir analysis. Gene expression data on sorted LT-HSCs (Lin-, CD34^+^, CD38^−^, CD90^+^), ST-HSCs (Lin-, CD34^+^, CD38^−^, CD90^−^), GMPs (Lin-, CD34^+^, CD38^+^, CD123+, CD45RA+) from patients with AML/MDS and healthy controls is deposited in the GEO database (GSE35008 and GSE35010).

#### FISH analysis of the RPL22 locus

A FISH probe for *1p36.2* with the RP11-MI719 BAC was produced commercially by Empire Genomics by nick translation. The TelVysion orange 1q probe was used as a control. Control values for the percent deletion of *RPL22* were established with pooled XY control bone marrow samples (pool of 20 bone marrows which tested negative by New York State and College of American Pathologists (CAP) Guidelines) as well as individual patient samples (patients with anemia and initial lymphoma bone marrow samples – all with normal cytogenetics). The probe was tested in 112 patient samples including patients with low risk MDS, high risk MDS, as well as primary and secondary AML. Over 2600 interphase cells in total were counted for the XY Control samples. At least 200 interphase cells were counted for each of the patient control samples as well as the MDS and AML samples. Metaphase cells were examined when identified. PRISM (http://www.graphpad.com/scientific-software/prism/) software was used to analyze the data for correlations with disease severity and other chromosomal abnormalities.

#### Complete blood counts

Peripheral blood was collected into EDTA coated tubes by cardiac puncture. Blood was analyzed using the Abaxis VetScan Hematology Analyzer (Union City, CA) according to the manufacturer’s instructions.

#### Immunofluorescence, histology, and immunoblotting

Sternums were formaldehyde fixed, decalcified, and subjected to H&E staining using a standard weak acid protocol. Tissues were paraffin embedded for sectioning. Immunofluorescence analysis was performed on bone marrow sections, bone marrow touch preps, or cytospun cell suspensions. Images were captured using a Nikon E800 upright microscope with BioRad Radiance 2000 confocal scanhead. Primary antibodies were conjugated using Alexa Fluor 594 or 488 (Life Technologies) and DAPI was used for nuclear staining. Rabbit polyclonal anti-Rpl22 and anti-Rpl22L1 antibodies were produced by conjugating the N-terminal 12 amino acids of Rpl22 (MAPVKKLVAKGG) and the C-terminal 12 amino acids of Rpl22L1 (ISQDEDESESED) to KLH and immunizing rabbits.^140^ Harvested anti-serum was assessed for specificity using samples from *Rpl22*^−/−^ and *Rpl22L1*^−/−^ mice. Immunoblotting was performed as described.^21^

#### Flow cytometric analysis and cell sorting of hematopoietic stem and progenitors

Bone Marrow was isolated either by flushing or crushing bones with mortar and pestle. Phenotypic analysis was performed using femurs only. The suspension was passed through a 100 μm filter, subjected to ACK Lysis (pH 7.4) of RBC, and then washed with PBS (without Ca2+ or Mg2+) containing 2% FBS. Enrichment for stem and progenitors was performed by negative selection of mature cells using rat antibodies to mature lineage markers (Gr-1, CD11b, B220, Ter119, and CD3) and goat anti-rat magnetic beads (Qiagen). Cells were stained using standard approaches with the indicated antibodies. PI or DAPI was used to exclude dead cells or for nuclear staining in cell cycle analysis. In assays requiring CD34 staining, cells were stained on ice for a minimum of one hour to ensure appropriate antigen binding. Cells were analyzed for flow cytometry using a BD LSR II and sorted using BD FACSAria II.

#### *In vivo* proliferation analysis of the hematopoietic compartment

Proliferation was assessed by BrdU incorporation. Briefly, mice were injected with BrdU at a dose of 0.15mg/g body weight. Mice were also maintained on water supplemented with 1mg/mL BrdU and 2% sucrose. After 24 h, mice were sacrificed and bone marrow was isolated. Cells were surfaced stained then fixed and permeabilized for nuclear staining using the Foxp3 Transcription Factor Staining Buffer Set (eBioscience). Cells were DNAse treated at 37°C for 1h and then incubated with anti-BrdU antibody (BioLegend). Cell cycle activity was also confirmed by Ki-67 Staining. Briefly, cells were lineage reduced then stained with a fixable live-dead dye. Lineage reduced cells were then surface stained for HSC markers, fixed, permeabilized using the Foxp3 Transcription Factor Staining Buffer Set, and then stained with anti-Ki-67, (BioLegend), following which they were analyzed by flow cytometry.

#### Competitive transplantation

CD45.1 mice were lethally irradiated with a total dose of 11 Gy, split in two doses of 6.5Gy and 4.5Gy, separated by three hours. After 24 h, mice were injected retro-orbitally with 300 CD45.2+ LSK/CD48−/CD150+ HSC and 200,000 CD45.1+ competitor bone marrow cells delivered in 200μL Hank’s Balanced Salt Solution (HBSS). Engraftment was monitored by retro-orbital bleeding every 4 weeks. Mice were sacrificed after 20 weeks of engraftment and bone marrow was analyzed by flow cytometry.

#### *In vivo* analysis of protein synthesis

Protein synthesis by HSC *in vivo* was measured with OP-Puro, as previously described.^43,44^ Mice were injected with 50mg/kg OP-Puro and after one hour the mice were sacrificed and bone marrow was isolated. After lineage reduction, cells were stained with GhostDye710, a fixable live/dead dye (Tonbo Biosciences), and analyzed by flow cytometry using the indicated antibodies. After staining, the cells were fixed and permeabilized using a Fix/Perm kit (BD Biosciences), following which the OP-Puro was conjugated to azide-linked Alexa Fluor 488 (Life Technologies) using the Click-It Cell Reaction kit (Life Technologies). Alexa 488 fluorescence was then measured by flow cytometry.

#### RNA-seq analysis

HSC were sorted directly into 500μL TriReagent (Sigma-Aldrich), as previously described, following which RNA was isolated according to the manufacturer’s protocol. RNA-Seq libraries were prepared using TruSeq RNA sample kit according to the manufacturer. The RNA-Seq gene set (Table S1) lists mRNA targets differential expressed between *Rpl22*^−/−^ and *Rpl22*^+/+^ HSC. RNA-Seq reads were processed for quality issues using FastQC (S.Andrews,http://www.bioinformatics.babraham.ac.uk/projects/fastqc/). Processed reads were aligned to the mouse genome (mm10) using TopHat2 (^141^, following which gene counts were quantified using HTSeq.^142^ The resulting gene counts were used as input for differential expression analysis between Rpl22^+/+^ and Rpl22^−/−^ HSC using DESeq2^143^ ). The application of a *p*p-value filter of 0.001 resulted in identification of 649 differentially expressed genes. The set of differentially expressed genes was then evaluated using the Mouse Genome Atlas, KEGG, and OMIM Disease Databases through the Enrichr Platform (http://amp.pharm.mssm.edu/Enrichr/.) To compare the genes differentially expressed in HSC upon Rpl22 loss to those modulated by MLL-AF9 expression, we compared our transcriptome data to that published by Stavropoulou et al.,^144^ focusing on the 72 h time point (GSE65384). The expression data was RMA normalized and Limma was used to identify differentially expressed genes.^145,146^ All calculations were done using packages from Bioconductor in the R programming environment.^147^ Heatmaps were generated using the Pheatmap package available through Bioconductor package repository. The RNA-Seq study was deposited in the Gene Expression Omnibus database (GSE237505).

For RNA-Seq on MLL-AF9 transgenic leukemias, explanted CD11b+Gr1+ splenocytes from tumor bearing *Rpl22+/+* and *Rpl22*^−/−^ MLL-AF9 transgenic mice were purified by flow cytometry and processed for RNA isolation using the NucleoZOL reagent (Macherey-Nagel), following the manufacturer’s protocol. RNA (250 ng) underwent poly(A) enrichment, first- and second-strand cDNA synthesis, and library amplification with the Illumina mRNA Stranded Library Kit, per the manufacturer’s instructions. Libraries were pooled, quantified, and loaded at 750 pM onto a NextSeq 2000 flow cell. Sequencing was performed with a 59–10-10–59 (R1-I1-I2-R2) cycle configuration, targeting 30–50 million paired-end reads per sample. Raw reads were trimmed with Trimmomatic to remove Illumina adaptors, and transcript quantification was conducted using Salmon.^148^ Downstream analysis—including principal component analysis and differential expression testing—was performed in R using DESeq2. Pathway and gene set enrichment analyses were carried out with EnrichR and GSEA. Plots were generated with ggplot2. The RNA-Seq data were deposited in the Gene Expression Omnibus database (GSE302046). CRISPR dependency scores were obtained from the DepMap portal.^149^ Lin28b targets were defined based on CLIP-seq data,^76^ and predicted *let-7–5p* targets were retrieved from TargetScan Mouse 8.0.^77^

#### Metabolic profiling

100,000 LSK cells were incubated overnight in the presence of C14-palmitate (1.7 μCi), TPO (100 ng/mL), and SCF (100 ng/mL), following which the cells and supernatant were acid precipitated to remove and non-oxidized palmitate. Soluble, oxidized lipid was quantified by liquid scintillation counting. FAO was also assessed on 150,000 MLL-AF9 transformed LSK labeled for 4h. Cellular bioenergetics of LSK cells sorted from Lin-depleted bone marrow cells was determined using the extracellular flux analyzer (XF^96^ analyzer, Seahorse Bioscience). Briefly, Seahorse cell culture analysis plates were coated with CellTak (BD Biosciences) one day before the experiment. LSK and MLL-AF9 transformed LSK cells were suspended in sterile serum-free assay buffer (RPMI1640 supplemented with 5.5 mM D-glucose, 4 mM L-glutamine, and 1 mM pyruvate, pH 7.4) and centrifuged for 5 min at 1200 rpm to allow them to settle and adhere on the plates. Media was carefully aspirated without disturbing the cells. Fresh assay media was added to control cells. Cells were treated with Etomoxir (100 μM) for 30 minutes at 37°C and then analyzed by the extracellular flux analyzer according to the manufacturer’s instruction. FCCP, Oligomycin, Rotenone/Actinomycin were injected in the wells following standard protocol of the Agilent Seahorse XF Cell Mito Stress Test.

#### Rpl22 regulation of Alox12 expression

Rpl22 binding sites in mRNA encoding regulators of FAO were identified as described using M-fold software with previously identified stem-loop binding sequence structures.^37^ To evaluate predicted binding sites in *Alox12* mRNA, EMSA analysis was performed. 35 nucleotide RNA oligos encompassing binding sites and controls were 5′-end labeled with γ-32P-ATP. 15pmol of each RNA oligo was incubated with 40μCi of γ-32P-ATP and 10U T4 polynucleotide kinase (NEB, M0201S) in 1x T4 polynucleotide kinase buffer (NEB) for 40 min at 37°C. Free ATP was removed using a NucAway spin column (ThermoFisher, AM10070). For EMSA reactions, 5nM of radioactive labeled RNA was added to binding buffer (37.5 mM HEPES (pH 7.9), 75 mM NaCl, 5 mM MgCl2, 0.1mg/ml BSA, 8% glycerol, and 1 μg E.coli tRNA) containing GST-Rpl22 fusion proteins. RNA-binding was assessed by electrophoresis on a non-denaturing gel. Oligo sequences used were as follows:

*Alox12* BS1: AUCCUGCUGGAUGGAAUUCCAGCUAAUGUGAU.

*Alox12* BS2: AUUUCCUCACCAUGUGUGUUUUCACAUGCACU.

*Alox12* NBS: ACCAGAGUGAUGAUAUUGUGAGGGGAGACCCA.

EBER2: GCUCAGUGCGGUGCUACCGACCCGAGGUCAAG.

EBER1: GGUCCGUCCCGGGUACAAGUCCCGGGUGGUGA.

To evaluate the capacity of Rpl22 to regulate ALOX12 expression, the *Alox12* coding region (pLVX-*Alox12-mCherry)* was retrovirally transduced into the *Rpl22*^−/−^ MEF line (KOML3), following which the cells were transduced with either empty vector (pMiG) or *Rpl22* (pMiG-*Rpl22).* Subsequently, the capacity of Rpl22 to regulate ALOX12 protein and mRNA was assessed by immunoblotting and qRT-PCR, respectively, on doubly transduced (mCherry/GFP double-positive) cells. To determine the basis by which Rpl22 regulates ALOX12 expression, the aforementioned cells were treated with cycloheximide, following which detergent extracts were subjected to sedimentation on a linear sucrose gradient to separate free mRNA from that being translated in heavy polysomes, as described.^21^ The *Alox12* mRNA content in the free and polysome-associated mRNA pools was then quantified by qRT-PCR and that in Rpl22-expressing cells was normalized to control transduced cells, and the distribution of *Gapdh* mRNA. The capacity of Rpl22 binding sites to transfer Rpl22-responsiveness to a heterologous mRNA target was assessed by appending target sequences with a start codon and fusing them in frame to GFP to generate a biosensor, as described.^18,92^ The constructs were then cloned into pCS2 and transfected into KOML3 cells, following which the GFP+ cells were transduced with empty vector (pMiCherry) or Rpl22 (pMiCherry-*Rpl22).* The effect on GFP levels were assessed by quantifying the mean fluorescence intensity (MFI) using FlowJo software, and mRNA levels were quantified by qRT-PCR in isolated, double-transduced cells. The target sequences employed comprise 141bp fragment of mouse wild *Alox12* containing the Rpl22 binding hairpin loop (BS1-WT) fused in frame with GFP and subcloned into pCS2+. The following primer sets were used:

mAlox12_F: CAGGGATCCATGGGGGAGACCCAGAGCTGCAGGC;

mAlox12_R: TCAGAATTCTGCATGTGAAAACACACATGGTGAGGAAATC.

The mutant fragment was generated such that the hairpin loop is not formed using the following reverse primer:

mAlox12_mutR: TCAGAATTCTGCATGTCTAAACACACATGGTGAGGAAAT.

#### Generation of MLL-AF9 leukemia lines

LSK were sorted from Rpl22^+/+^ and Rpl22^−/−^ mice, transduced with MLL-AF9-retroviruses, and serially-passaged through M3434 methylcellulose until stabilized (StemCell Technologies). Cells were then maintained in IMDM supplemented with 10% FBS, 10 ng/mL SCF, 6 ng/mL IL-3, and 5 ng/mL IL-6.

#### Colony formation assays

For colony formation assays, 500 primary or MLL-AF9 transformed LSK were plated in M3434 Methylcellulose (Stem Cell Technologies) in 35 mm^2^ dishes. Colonies were counted every seven days. For serial replating assays, after seven days cells were isolated by dissolution in media and 10,000 cells were re-passaged. To ectopically express ALOX12, the murine *Alox12* coding sequence was subcloned into the pLVX-IRES-mCherry lentiviral vector. 48h after transduction into *Rpl22+/+* LSK, the mCherry+ cells were purified by cell sorting and subjected to colony formation analysis as above. *Ppard* knockdown in MLL-AF9 leukemias was performed using shRNA in pRFP-C-RS that were obtained from Origene:

shPPARd-1: AGGTAGAAGCCATCCAGGACACCATTCTG

shPPARd-4: AGCATCCTCACCGGCAAGTCCAGCCACAA.

RFP+ MLL-AF9 leukemias transduced with control (NT) or *Ppard-*targeting shRNA were sorted and then plated to assess the effect on colony formation as described above. For drug treatment with etomoxir, 500 primary LSK or MLL-AF9 transformed LSK cells were resuspended in M3434 Methylcellulose supplemented with 200 μM etomoxir. Cells were plated on 35 mm^2^ plates and colony formation was assessed after seven days. Etomoxir was purchased from Sigma-Aldrich and dissolved as specified by the manufacturer. The drug was stored in the dark at −20C, and thawed only once prior to use. For drug treatment with baicalein, 500 MLL-AF9 transformed LSKs were resuspended in M3434 methylcellulose supplemented with indicated concentrations of drug. Baicalein was purchased from Sigma-Aldrich and fresh dilutions were made prior to each experiment in DMSO. The drug was stored in the dark at −20C.

#### Metabolite quantitation

Levels of 12(S)-HETE were measured in triplicate in flow cytometrically sorted LSK from Rpl22^+/+^ and Rpl22^−/−^ mice. The cells were assayed for 12(S)-HETE levels using the 12(S)-HETE ELISA kit from Enzo Life Sciences, Inc (Ann Arbor, MI) according to the manufacturer’s instructions. 25,000 cells per well were loaded to assess 12(S)-HETE production and were normalized to 12(S)-HETE levels in Rpl22^+/+^ LSK. Lactate and ATP were measured using Colorimetric Assay Kits from Biovision (Cat#K607 and Cat#K354, respectively) according to manufacturer’s recommendations. For lactate measurements, LSK cells were lysed in hypotonic lysis buffer (10mM Tris-Cl (pH7.2), 1mM EDTA, 150 mM NaCl, Protease inhibitor, 0.05% Triton X-100), and then the clarified supernatant was used to quantify lactate at 570nm using a standard curve. For ATP measurement, purified LSK and lysed in ATP Assay Buffer, deproteinized using a 10kDa spin column, and used to quantify ATP using a standard curve.

#### Lin28b regulation of lipid content and survival

Explanted Rpl22^+/+^ and Rpl22^−/−^ leukemias from MLL-AF9 transgenic mice were cultured *in vitro* in 10ng/ml murine stem cell factor, 6ng/ml interleukin-6, and 5ng/ml interleukin-3. Lipid content of these cells was measured by staining with Nile Red. 5 ×10^6^ cells were stained in 1mL HBSS containing 3μM Nile Red at 37^o^C for 10 min, washed with cold HBSS containing 1%BSA, and assessed by flow cytometry using Helix Blue as a viability dye. In addition, triglycerides (TG) were measured in detergent extracts of Rpl22^+/+^ and Rpl22^−/−^ MLL-AF9 Tg leukemias using the Abcam TG Assay Kit according to manufacturer’s recommendations. The impact of Lin28b knockdown on lipid content and growth of leukemias was assessed by knocking down Lin28b using shRNA.

pLKO (Puromycin resistance cassette replaced with GFP):

NT – CCGGCAACAAGATGAAGAGCACCAACTCGAGTTGGTGCTCTTCATCTTGTTGTTTTTG

sh7 - CCGGGCCAGTGGAATTTACATTTAACTCGAGTTAAATGTAAATTCCACTGGCTTTTTG

sh9 - CCGGCGGCAGGATTTACTGATGGATCTCGAGATCCATCAGTAAATCCTGCCGTTTTTG.

Leukemia lines were transduced with the shRNA encoding lentiviral constructs and 48h later GFP-expressing transduced cells were isolated by flow cytometry. Isolated cells were used for the analysis above. The impact of Lin28b shRNA on *Lin28b* expression was assessed by quantitative PCR using a Taqman probe set (Mm01190673_m1; ThermoFisher). The dependence of *Rpl22+/+* and *−/−* leukemias on TG was determined by performing MTT assays according to the manufacturer’s recommendations following treatment with a DGAT1 inhibitor (DGAT1-IN-1).

### QUANTITATION AND STATISTICAL ANALYSIS

Details of statistical methods are found in the legends of each figure. Statistical significance was assessed using a variety of methods. Statistical significance between groups of graphed data was determined using the Student’s *t* test unless specified otherwise with triplicate measured expressed graphically as the mean ± standard error of the mean (SEM). In ScatterPlots, each analyte (i.e., individual mice or patients with myeloid disease) is denoted by an individual symbol. ROUT method was used to identify and confirm clear outliers. Outliers determined by this method are denoted using an asterisk on graphs. Survival-Curves were analyzed using the Mantel-Cox log rank test. All analyses were performed using Microsoft Excel or GraphPad Prism Software. All experiments were conducted a minimum of three times. P-values, Z-scores, or combined scores associated with gene set enrichment analyses were obtained using the built-in function used by the Enrichr Analysis.

## Supplementary Material

1

2

3

4

5

6

SUPPLEMENTAL INFORMATION

Supplemental information can be found online at https://doi.org/10.1016/j.celrep.2025.116688.

## Figures and Tables

**KEY RESOURCES TABLE:** 

REAGENT or RESOURCE	SOURCE	IDENTIFIER
Antibodies		
Polyclonal rabbit anti-Alox12	Boster Bio	A02275-1
Polyclonal rabbit anti-PPAR5	Invitrogen	PA1-823A
Polyclonal rabbit anti-Rpl22	This lab; this report	Custom
Polyclonal rabbit anti-Rpl22L1	This lab; this report	Custom
Rabbit monoclonal anti-AMPK	Cell Signaling	5832S
Rabbit monoclonal anti-pAMPK	Cell Signaling	–
Mouse monoclonal anti-tubulin	Santa Cruz Biotech.	Sc-5286 HRP
Mouse monoclonal anti-GAPDH	EMD Millipore Corp.	CB1001-500ug
Biological samples		
Murine HSC; Lin-Kit+Sca1+CD150+CD48^−^	This lab, this study	Kiel et al.^33^
Murine LSK; Lin-Kit+Sca1+	This lab, this study	Oguro et al.^44^
Leukapheresed CD34+ human primary AML/MDS	This lab, this study	Choudhary etal.^136^
Chemicals, peptides, and recombinant proteins		
Rpl22 N-terminal peptide – MAPVKKLVAKGG	Alpha Diagnostics	Custom
Rpl22L1 C-terminal peptide - ISQDEDESESED	Alpha Diagnostics	Custom
OP-puromycin	Jena Biosciences	NU-931-5
DGAT1 inhibitor - DGAT1-IN-1	MedChemExpress	HY-12425
Critical commercial assays		
Seahorse XF Cell Mito Stress Test	Agilent	103595–100
TruSeq RNA sample kit	Illumina	S-122-2001
12(S)-HETE ELISA kit	Enzo	ADI-900-050
Click-iT Cell Reaction Buffer Kit	Invitrogen	C10269
Triglyceride Assay Kit	Abcam	ab65336
Deposited data		
RNA-Seq on *Rpl22*+/+ and *Rpl22*^−/−^ HSC	GEO	GSE237505
RNA-Seq on LT-HSCs (Lin-, CD34^+^, CD38 , CD90^+^), ST-HSCs (Lin-, CD34^+^, CD38^−^, CD90^−^), GMPs (Lin-, CD34^+^, CD38^+^, CD123^+^, CD45RA^+^) from patients with AML/MDS and healthy controls	GEO	GSE35008 and GSE35010
RNA-Seq on 183 MDS and 17 healthy CD34^+^ control bone marrow samples	GEO	GSE19429
RNA-Seq on CD11b+Gr1+ MLL-AF9 leukemias from Rpl22^+/+^ and Rpl22^−/−^ mice	GEO	GSE302046
Experimental models: Cell lines		
*In vitro* transformed MLL-AF9 *Rpl22*+/+ and −/− leukemia lines	This lab, this study	Sykes et al.^137^
*Ex vivo* MLL-AF9 *Rpl22*+/+ and −/− leukemia lines	This lab, this study	Ballabio et al.^138^
Experimental models: Organisms/strains		
Rpl22^−/−^ mice	Anderson et al.^19^	PMID: 17555992
Rpl221fl/fl VavCre mice	Fahl et al.^139^	PMID: 35046107
MLL-AF9 knockin mice	Corral et al., 1996	PMID: 8681380
Alox12^−/−^ mice	Sun and Funk^55^	PMID: 8798642
Oligonucleotides		
*Alox12* BS1: AUCCUGCUGGAUGGAAUUCCAGCUAAUGUGAU	IDT	Custom
*Alox12* BS2: AUUUCCUCACCAUGUGUGUUUUCACAUGCACU	IDT	Custom
*Alox12* NBS: ACCAGAGUGAUGAUAUUGUGAGGGGAGACCCA	IDT	Custom
EBER2 – GCUCAGUGCGGUGCUACCGACCCGAGGUCAAG	IDT	Custom
EBER1 -GGUCCGUCCCGGGUACAAGUCCCGGGUGGUGA	IDT	Custom
mAlox12_F: CAGGGATCCATGGGGGAGACCCAGAGCTGCAGGC	IDT	Custom
mAlox12_R: TCAGAATTCTGCATGTGAAAACACACATGGTGAGGAAATC	IDT	Custom
mAlox12_mutR: TCAGAATTCTGCATGTCTAAACACACATGGTGAGGAAAT	IDT	Custom
pLKO-NT: CCGGCAACAAGATGAAGAGCACCAACTCGAGTT GGTGCTCTTCATCTTGTTGTTTTTG	Sigma	Custom
pLKO-Lin28b sh7 CCGGGCCAGTGGAATTTACATTTAACTCGAGTTAAATG TAAATTCCACTGGCTTTTTG	Sigma	TRCN0000124705
pLKO-Lin28b sh9 CCGGCGGCAGGATTTACTGATGGATCTCGAGATCCAT CAGTAAATCCTGCCGTTTTTG	Sigma	TRCN0000124707
Lin28b Taqman Primer/Probe set	ThermoFisher	Mm01190673_m1
Recombinant DNA		
pRFP-C-RS shPPARd-1 -AGGTAGAAGCCATCCAGGACACCATTCTG	Origene	Custom
pRFP-C-RS shPPARd-4 - AGCATCCTCACCGGCAAGTCCAGCCACAA	Origene	Custom

## Highlights

- RPL22 insufficiency is observed in MDS/AML and is associated with reduced survival
- Rpl22 deficiency produces an MDS-like syndrome and facilitates leukemogenesis
- Rpl22 deficiency does not impair global protein synthesis by HSCs
- Rpl22 controls leukemia survival through control of lipid synthesis
