# Supplementary material for: Ribosomal protein control of hematopoietic stem cell transformation through regulation of metabolism
Source: Cell Rep. Author manuscript; Available in PMC 2026 Jan 21. (PMC12820725; doi:10.1016/j.celrep.2025.116688)
Supplement: 6 [file NIHMS2132768-supplement-6.pdf]

Antigen	Clone	Fluorophore	Company
B220	RA3-6B2	APC-Cy7	Biolegend
B220	RA3-6B2	FITC	Biolegend
B220	RA3-6B2	Purified	Biolegend
CD11b	M1/70	PE-Cy7	Biolegend
CD11b	M1/70	FITC	Biolegend
CD11b	M1/70	RF710	Tonbo Bio
CD11b	M1/70	APC-Cy7	Biolegend
CD11b	M1/70	Purified	Biolegend
CD150	TC15-12F12.2	BV421	Biolegend
CD16/32	2.4G2	BV605	BD Bioscience
CD16/32	93	PE	Biolegend
CD16/32	93	Alexa Fluor 700	eBioscience
CD19	eBio1D3	FITC	eBioscience
CD3	145-2C11	FITC	Biolegend
CD3	17A2	RF710	Tonbo Bio
CD3	145-2C11	PE-TR	BD Bioscience
CD3	17A2	APC-Cy7	Biolegend
CD3	17A2	Purified	Biolegend
CD34	RAM34	Biotin	eBioscience
CD34	RAM34	eFluor660	eBioscience
CD41	ebioMWReg30	PE	eBioscience
CD41	MWReg30	APC	Biolegend
CD45.1	A20	PE	BD Bioscience
CD45.1	A20	APC-Cy7	Biolegend
CD45.2	104	RF710	Tonbo Bio
CD45.2	104	APC-Cy7	Biolegend
CD48	HM48-1	PerCP-Cy5.5	Biolegend
CD48	HM48-1	BV421	Biolegend
c-Kit	2B8	APC	Biolegend
c-Kit	2B8	APC-eFluor 780	eBioscience
Gr-1	RB6-8C5	Alexa Fluor 700	Biolegend
Gr-1	RB6-8C5	FITC	Biolegend
Gr-1	RB6-8C5	RF710	Tonbo Bio
Gr-1	RB6-8C5	APC-Cy7	Biolegend
Gr-1	RB6-8C5	Purified	Biolegend
Ki-67	16A8	FITC	Biolegend
M-CSFR	AFS98	Biotin	Biolegend
SA-Biotin		QDOT605	Life Tech
Sca-1	D7	PE-Cy7	Biolegend
Sca-1	D7	PE	BD Bioscience
TER119	TER119	FITC	Biolegend
TER119	TER119	PerCP-Cy5.5	Biolegend
TER119	TER119	APC-Cy7	Biolegend
TER119	TER119	RF710	Tonbo Bio
TER119	TER119	PE-Cy5	Biolegend
TER119	TER119	Purified	Biolegend
